# Taraxasterol Inhibits Tumor Growth by Inducing Apoptosis and Modulating the Tumor Microenvironment in Non-Small Cell Lung Cancer

**DOI:** 10.3390/cancers14194645

**Published:** 2022-09-24

**Authors:** Junjie Lu, Bo Shuai, Zhexing Shou, Weina Guo, Cong Zhou, Xiaohu Ouyang, Haifeng Zhou, Junyi Li, Jing Cui, Feng Jiang, Kim Yun Jin, Alexey Sarapultsev, Fangfei Li, Ge Zhang, Shanshan Luo, Desheng Hu

**Affiliations:** 1 Department of Integrated Traditional Chinese and Western Medicine, Union Hospital, Tongji Medical College, Huazhong University of Science and Technology, Wuhan 430022, China; 2 Department of Laboratory Medicine, Wuhan Children’s Hospital (Wuhan Maternal and Child Healthcare Hospital), Tongji Medical College, Huazhong University of Science and Technology, Wuhan 430015, China; 3 Health Management Center, Hubei Provincial Hospital of Integrated Chinese & Western Medicine, Wuhan 430010, China; 4 College of International Education, Tianjin University of Traditional Chinese Medicine, Tianjin 301617, China; 5 School of Traditional Chinese Medicine, Xiamen University Malaysia, Sepang 43900, Malaysia; 6 Russian-Chinese Education and Research Center of System Pathology, South Ural State University, 454000 Chelyabinsk, Russia; 7 Shum Yiu Foon Sum Bik Chuen Memorial Centre for Cancer and Inflammation Research, School of Chinese Medicine, Hong Kong Baptist University, Hong Kong 999077, China; 8 Institute of Integrated Bioinfomedicine and Translational Science, School of Chinese Medicine, Hong Kong Baptist University, Hong Kong 999077, China; 9 Institute of Hematology, Union Hospital, Tongji Medical College, Huazhong University of Science and Technology, Wuhan 430022, China; 10 Key Laboratory of Biological Targeted Therapy, The Ministry of Education, Wuhan 430022, China; 11 Clinical Research Center of Cancer Immunotherapy, Union Hospital, Tongji Medical College, Huazhong University of Science and Technology, Wuhan 430022, China

**Keywords:** TAX, NSCLC, EMT, apoptosis, immune cells

## Abstract

**Simple Summary:**

Taraxasterol (TAX) demonstrates strong pharmacological activity in some diseases. In this study, we demonstrate that TAX induces S-phase cell cycle arrest, prevents cell migration by interfering EMT, and induces cancer cell apoptosis. In addition, TAX administration downregulated the proportion of Treg cells and upregulated CD107a + NK cells in TME. Our in vitro and in vivo findings indicate that TAX could serve as a potential natural drug for lung cancer therapy.

**Abstract:**

Taraxasterol (TAX), one of the active components in Dandelion, demonstrated strong antitumor properties in several cancers. However, the effect and underlying mechanism of TAX in non-small cell lung cancer (NSCLC) is unclear. In this study, we showed that TAX inhibited the proliferation of cells by inducing S-phase cell cycle arrest and prevented cell migration by interfering epithelial-mesenchymal transition (EMT) in Lewis lung cancer (LLC) cells and lung carcinoma SPC-A1 cells. The pharmacological network analysis predicted that induction of apoptosis might be the potential mechanism of TAX-mediated cell deaths. Further in vitro experiments showed that TAX could significantly induce cancer cell apoptosis as verified by increased pro-apoptotic molecules including Bax, caspase-9, and PARP1 downregulated anti-apoptotic protein Bcl-2; and decreased mitochondrial potential. The LLC subcutaneous tumor model demonstrated that TAX inhibited tumor growth by induction of apoptosis and inhibition of proliferation in vivo, which is consistent with the in vitro data. Importantly, TAX administration downregulated the proportion of Treg cells and upregulated CD107a+ NK cells in the tumor microenvironment in the tumor model. Together, these data reveal that TAX performs its antitumor effect by inducing apoptosis and modulating the tumor microenvironment, providing evidence that TAX could serve as a potential natural drug for lung cancer therapy.

## 1. Introduction

Lung cancer is the number one cause of cancer-related death worldwide [1]. More than 85% of lung cancers are staged as non-small cell lung cancer (NSCLC). Although great advances in lung cancer therapy have been made, both mortality and morbidity still remain at high levels, second only to those of cardiovascular diseases, and the global cancer burden is huge and growing [2]. Surgery, chemotherapy, radiotherapy, and immunotherapy are the common therapies for NSCLC [3]. However, low physical condition of patients, obvious side effects, as well as high medical expenditure are still the challenges of current treatment. Therefore, an alternative drug or therapy is urgently needed to improve the treatment of NSCLC patients.

Apoptosis is a well-recognized process of programmed cell death and a key part of the innate tumor-suppression mechanism. Inducing apoptosis of tumor cells is considered a promising option, and many drugs and therapeutic measures are designed to exert efficient anti-tumor effects by inducing apoptosis of tumor cells. Upregulation of the proapoptotic protein Bax and caspase cascade, and downregulation of the antiapoptotic proteins Bcl-2, Bcl-xL are regarded as the central steps for controlling intrinsic apoptosis. Clinical trials have shown that venetoclax, a drug targeting Bcl-2, substantially reduced the tumor burden at a certain dose range [4]. However, drug resistance arises along with the application of apoptosis-inducing therapies, leading to reduced efficacy in the clinic; therefore, intensive mechanistic study is needed.

The tumor microenvironment (TME) is a complex and integrated system formed by the interaction of tumor cells with surrounding stromal cells, myeloid cells, and immune cells [5]. TME facilitates the proliferation and migration of tumor cells, and promotes immune escape and immune tolerance; and thus impels the progression of tumors [6,7]. Therefore, modulation of TME, including targeting tumor metabolism to inhibit tumor cell growth and effector cell metabolism to enhance the killing ability of tumors, is an extremely promising therapeutic strategy in tumor treatment.

Dandelion, a Chinese medicinal herb, belonging to the dandelion genus of the Asteraceae family, possesses anti-inflammation [8,9], anti-bacterial [10], and anti-tumor effects [11,12]. The extraction of dandelion inhibited the proliferation, migration, and invasion of breast cancer cells by suppressing the IL-10/STAT3/PD-L1 immunosuppressive signaling pathway, and promoted macrophage differentiation from the M2 to M1 phenotype [13]. Taraxasterol (TAX) is one of the main active ingredients of dandelion. Studies showed that TAX prevented the growth of melanoma cells by inhibiting the reactive oxygen species-mediated PI3K/Akt signaling pathway [14]. Studies also revealed that TAX exerts anti-colorectal cancer effects by promoting autophagy and oncogene NRF31 degradation in colorectal cancer, and inhibits Epithelial-mesenchymal transition (EMT) through regulating the Wnt/β-catenin signaling pathway in thyroid cancer [15]. However, the effects and concrete mechanisms of TAX on NSCLC have not been clarified [16].

In this research, we verified the antitumor effect of TAX and investigated its underlying mechanisms. Our data indicated that the natural product TAX inhibited migration and proliferation of LLC and SPC-A1 cells, promoted tumor cell apoptosis, and modulated TME in vivo. Our findings suggest that TAX could be a promising compound for the treatment of NSCLC.

## 2. Materials and Methods

### 2.1. Cell Lines and Reagents

SPC-A1 LLC cell lines were donated by the Department of Respiratory and Critical Care Medicine, Wuhan Union Hospital, Tongji Medical College, Huazhong University of Science and Technology. The LLC cells were maintained in DMEM/high glucose medium (HYCEZMBIO, Wuhan, China), the SPC-A1 cells were maintained in RPMI-1640 medium (HYCEZMBIO, Wuhan, China), and all cell lines were maintained in the medium with 10% fetal bovine serum (FBS) and 1% penicillin/streptomycin. The cells were placed in a cell culture incubator with a humidified atmosphere of 95% air and 5% CO_2_ at 37 °C. TAX was purchased from Chengdu Herbpurify Co. Ltd. (Chengdu, China), and its purity was 98% based on HPLC analysis. TAX was dissolved in absolute ethyl alcohol (Sinopharm Chemical Reagent Co. Ltd., Shanghai, China) at 5 mM just before use. Different groups of cells were treated as follows: the control group (no processing) and TAX treatment group (with different concentrations of TAX).

### 2.2. CCK-8 Assay

The Cell Counting Kit-8 (CCK-8) (GlpBio Technology, Montclair, CA, USA) was used for the determination of cell viability according to the instructions. LLC and SPC-A1 cells were seeded in 96-well plates at a density of 3 × 10^3^ cells/well and incubated with TAX at different concentrations for the indicated time periods at 37 °C. After different intervention times, 10 μL CCK-8 solution was added into the culture and incubated at 37 °C for 1 h. The absorbance was measured using a microplate reader (Molecular Devices, San Jose, CA, USA) at 450 nm. Details can be found in reference [17]. Further details are also shown in the Supplementary Materials.

### 2.3. LDH Release Assay

The LDH Cytotoxicity Assay Kit (Beyotime Biotechnology, Shanghai, China) was used for the determination of cellular LDH release according to the instructions. LLC and SPC-A1 cells were seeded in 96-well plates at a density of 3 × 10^3^ cells/well and incubated for the indicated time periods at 37 °C. After different intervention times, the cell culture plates were centrifuged at 400× *g*, 5 min. Next, 120 μL supernatant of each well was taken into a new 96-well plate. In each well, 60 μL LDH assay working solution was added and incubated for 30 min at room temperature in the darkness. The absorbance was measured using a microplate reader (Molecular Devices, San Jose, CA, USA) at 492 nm. Details can be found in reference [18]. Further details are also shown in the Supplementary Materials.

### 2.4. Cell Cycle Assay

The Cell Cycle Staining Kit (Multi Sciences Biotech, Zhejiang, China) was used for determination of the cell cycle according to the instructions. LLC and SPC-A1 cells (3 × 10^5^ cells/well) were seeded in 24-well plates for 24 h and treated with TAX at different concentrations for another 24 h. Cells were harvested and washed with precooling PBS twice, and then stained with 1 mL DNA staining solution and 10 μL permeabilization solution at room temperature for 30 min in the darkness. After washing, the cells were measured and analyzed by flow cytometry (CytoFLEX, Backman Counter, Brea, CA, USA). Details can be found in reference [17]. Further details are also shown in the Supplementary Materials.

### 2.5. Measurement of the Cell Apoptosis

Apoptosis was assessed using the annexin V/7-AAD apoptosis kit (Multi Sciences Biotech, Zhejiang, China) according to the instructions. LLC and SPC-A1 cells (5 × 10^4^/well) were seeded in 24-well plates for 24 h and treated with TAX for 48 h. The cells were harvested and washed with precooling PBS twice, then incubated with annexin V and 7-AAD dyes at room temperature for 5 min in darkness. The stained cells were measured and analyzed by flow cytometry (CytoFLEX, Backman Counter, Brea, CA, USA). Details can be found in reference [17]. Further details are also shown in the Supplementary Materials.

### 2.6. Measurement of ROS

The Reactive Oxygen Species Assay Kit (Beyotime Biotechnology, Shanghai, China) was used for the determination of ROS according to the instructions. SPC-A1 cells were seeded in 24-well plates at a density of 5 × 10^4^ cells and treated with TAX for 48 h. Cells were washed with precooling PBS twice and incubated with 10 μM DCFH-DA for 20 min within a 37 °C cell incubator. The cells were washed three times with serum-free cell culture solution to fully remove free DCFH-DA probes. DCFH-DA was detected and analyzed by flow cytometer (CytoFLEX, Backman Counter, CA, USA). Details can be found in reference [17]. Further details are also shown in the Supplementary Materials.

### 2.7. Mitochondrial Membrane Potential Detection

An enhanced mitochondrial membrane potential assay kit with JC-1(Beyotime Biotechnology, Shanghai, China) was used for detecting mitochondrial membrane potential changes according to the instructions. SPC-A1 cells (1 × 10^6^/well) were seeded in 6-well plates and treated with TAX for 48 h at 37 °C. Cells were harvested and washed with precooling PBS twice. Then, the cells were suspended in JC-1 staining working solution and incubated for 20 min at 37 °C in a cell incubator. After being washed with JC-1 staining buffer twice, the stained cells were observed under a fluorescence microscope (Olympus-life science, Tokyo, Japan), and mean fluorescence intensity was quantified by Image J software (V1.53k, National Institutes of Health Maryland, Bethesda, MD, USA) Details can be found in reference [19]. Further details are also shown in the Supplementary Materials.

### 2.8. Wound-Healing Assay

For the Transwell migration assay, LLC and SPC-A1 cells were cultured to full confluence. Then, we artificially created a wound using a sterile plastic pipette tip. After being washed with PBS twice, the medium was replaced with serum-free medium containing TAX at different concentrations and incubated at 37 °C for 36 h. After being washed twice with PBS, the wound was observed under a microscope (CX53, Olympus-life science, Tokyo, Japan).

### 2.9. Colony-Formation Assay

For the colony-formation assays in monolayer cultures, LLC and SPC-A1 cells (150 cells/well) were seeded in 100 mm dishes for 24 h and then treated with TAX at different concentrations for 10 days. Then, the cells were fixed in 4% paraformaldehyde (Servicebio, Wuhan, China) at 4 °C and stained with crystal violet (Biosharp, Hefei, China). Details can be found in reference [17]. Further details are also shown in the Supplementary Materials.

### 2.10. Western Blot Analysis

The LLC cells and SPC-A1 cells (1 × 10^6^ cells/well) were seeded in 6-well plates for 24 h and treated with different concentrations of TAX for another 48 h. The cells were washed with PBS twice and lysed by RIPA Lysis Buffer (Servicebio, Wuhan, China) with the addition of Phenylmethanesulfonyl fluoride to protect the protein from degradation (Servicebio, Wuhan, China). Protein loading buffer was added, and protein denaturation was carried out at 95 °C for 15 min. The protein samples were separated by 10 or 12% twelve alkyl SDS-PAGE and transferred onto a PVDF membrane (Merck Millipore, Darmstadt, Germany). The PVDF membrane was blocked with 5% milk in TBST powder for 1 h and incubated with the primary antibody (β-actin, Servicebio, Wuhan, China; Snail1/Snail2/Vimentin/Caspase-9, ABclonal, Wuhan, China; N-cadherin/E-cadherin/Bax/Bcl-2/PAPR1/Cytochrome C, ProteinTech, Wuhan, China) at 4 °C overnight. The membranes were washed with TBST (three times × 15 min), then incubated with HRP conjugated secondary antibody at room temperature for 1.5 h. After being washed with TBST (three times × 15 min), the membranes were incubated with ECL solution (Biosharp, Hefei, China), and images were obtained with the iBright CL750 imaging system (ThermoFisher Scientific, Waltham, MA, USA). Protein levels were quantified by Image J software. Details can be found in reference [20]. Further details are also shown in the Supplementary Materials.

### 2.11. mRNA Expression Analysis

SPC-A1 cells were seeded in 6-well plates at a density of 1 × 10^6^ cells and incubated with TAX at different concentrations for 48 h. After the cells were harvested, total RNA was extracted with Trizol (Takara Biomedical Technology Co., Ltd., Shiga, Japan) and Trichloromethane (Sinopharm Chemical Reagent Co., Ltd., Shanghai, China). Next, according to the instructions of the SynScript III cDNA Synthesis Mix kit (Tsingke Biotechnology, Beijing, China), the total RNA was reverse transcribed into cDNA. Then, the cDNA was amplified on the fluorescence quantitative PCR instrument (ABI StepOnePlus Real-Time PCR System with Tower, Waltham, MA, USA). Amplification reactions were performed according to the manufacturer’s protocol (Vazyme, 2 × Acetaq Master Mix dye plus, Nanjing, China) under the reaction conditions used for amplification. The calculation is based on the 2^−ΔΔCT^ method, and β-actin was used as a reference to normalize the expression and calculate the relative expression of each group. Table 1 shows the gene and primer sequences (Table 1). Details can be found in reference [21]. Further details are also shown in the Supplementary Materials.

### 2.12. Immunofluorescence

Immunohistochemistry was performed as previously reported. Briefly, frozen tissue sections were fixed and antigenically repaired. Afterward, tissue sections were incubated overnight at 4 °C with a mouse monoclonal anti-Ki-67 (1:100; Servicebio, Wuhan, China) followed by a secondary antibody. After rinsing in water, sections were counterstained with hematoxylin, dehydrated, and capped. Afterward, they were observed under a microscope and quantified by Image J software and the IHC Profiler plug-in.

### 2.13. TUNEL Fluorescence Detection

The TUNEL fluorescence assay was performed according to the instructions of the Fluorescein (FITC) Tunel Cell Apoptosis Detection Kit (Servicebio, Wuhan, China). Details can be found in reference [18]. Further details are also shown in the Supplementary Materials. The frozen sections of tumor tissue were fixed with 4% paraformaldehyde for about 30–60 min. Then, the fixed sections were washed 2 times with PBS for 10 min each, and PBS added containing 0.1% Triton X-100 and incubated in an ice bath for 2 min. Next, the sections were equilibrated, labeled, nuclear stained, and blocked. Finally, the sections were observed under a fluorescent microscope, and mean fluorescence intensity was quantified by Image J software.

### 2.14. Determination of Serum ALT, AST, Cre, and BUN in Mice

The peripheral venous blood of mice was collected, and serum was separated by centrifugation (4 °C, 3000 rpm 10 min). The expression of serum ALT, AST, Cre, and BUN was determined according to the instructions provided in the reagent kits (Nanjing Jiancheng Bioengineering Institute, Nanjing, China).

### 2.15. Detection of the Immune Microenvironment in Tumor

After pentobarbital anesthesia, the mice were killed by cervical dislocation, and the tumor tissue was stripped and cut into pieces, and then digested with 1 mg/mL collagenase IV (Sigma-Aldrich, Darmstadt, Germany) and 0.2 mg/mL DNase I (Sigma-Aldrich, Darmstadt, Germany) for 1 h at 37 °C. Red blood cells were lysed with Red Blood Cell Lysis Buffer (Servicebio, Wuhan, China) to eliminate the interference of red blood cell spontaneous fluorescence. Tissue scissors were used to cut tumor tissue to obtain the single-cell suspension. Single cell suspensions were stained with the indicated antibodies diluted by FACS buffer for surface markers. For the staining of intracellular cytokines IL-17a, IFN-γ, Perforin, and CD107a, the cells were incubated and stimulated with 200 ng/mL phorbol myristate acetate (PMA) (PMA, Santa Cruz Biotechnology, Texas, CA, USA), 1 μg/mL ionomycin (Enzo, Farmingdale, NY, USA), and 1 μg/mL brefeldin A (eBioscience, Carlsbad, Santiago, CA, USA) at 37 °C for 5–6 h. Next, cultured cells were collected, washed, and stained with surface markers for 15–30 min, and then stained intracellularly with anti-IL-17a, anti-IFN-γ, anti-Perforin, and anti-CD107a antibodies for 30–45 min after fixation and permeabilization. For Foxp3 staining, the cells were stained for surface markers such as CD45 and CD4, followed by fixation and permeabilization with fixation and permeabilization buffer (Thermo Fisher Scientific, Waltham, MA, USA) at room temperature for 30–45 min. After being washed, the cells were then stained with anti-Foxp3 antibody as instructed. Details can be found in reference [22,23,24]. Further details are also shown in Supplementary Materials. All samples were detected by the CytoFLEX LX Flow Cytometry System and analyzed with CytExpert 2.4 Software (V2.4, Backman Counter, CA, USA). Antibodies used for flow cytometry included anti-mouse CD45-FITC (BD Biosciences, Franklin Lakes, NJ, USA), CD3-APC-Cy7 (BioLegend, San Diego, CA, USA), CD4-PE-Cy7 (BioLegend, San Diego, CA), CD8-PerCP-Cy5.5 (BioLegend, San Diego, CA, USA), CD11b-APC-Cy7 (BioLegend, San Diego, CA, USA), CD11c-PE-Cy7 (BioLegend, San Diego, CA, USA), CD107a-BV421 (BioLegend, San Diego, CA, USA), CD206-APC (BioLegend, San Diego, CA, USA), F4/80-PE (BioLegend, San Diego, CA, USA), NK1.1-PE-Cy7 (BD Biosciences, Franklin Lakes, NJ, USA), MHC-II-PerCP-Cy5.5 (BD Biosciences, Franklin Lakes, NJ, USA), Perforin-PE (BD Biosciences, Franklin Lakes, NJ, USA), IFN-γ-APC (ThermoFisher Scientific, Waltham, MA, USA), IL-4-BV421 (ThermoFisher Scientific, Waltham, MA, USA), IL-17a-PE (BD Biosciences, Franklin Lakes, NJ, USA), FoxP3-PE (BD Biosciences, Franklin Lakes, NJ, USA), FVD-KO525 (ThermoFisher Scientific, Waltham, MA, USA), FVS- PerCP-Cy5.5 (ThermoFisher Scientific, Waltham, MA, USA).

### 2.16. In Vivo Tumor Model

All the in vivo experimental protocols were approved by the animal Nursing Committee of Tongji Medical College of the Huazhong University of Science and Technology. LLC cells (2 × 10^6^ cells in 0.1 mL phosphate-buffered saline) were subcutaneously injected into the dorsal side of 6-week-old male C57 mice. The tumor volume was evaluated every 2 days, and the weight of the mice every 3 days, and calculated by the following formula: (short diameter)^2^ × (long diameter)/2 [17]. After confirming the tumor formation, the mice were randomly divided into 4 groups: control group; low dose (5 mg/kg), medium dose (25 mg/kg), and high dose (100 mg/kg) group. Fifteen days after administration (gavage treatment, once every other day), the mice were killed by cervical dislocation, tumor tissue was stripped for photography and recording, blood samples and tissue samples were obtained for drug safety testing, and tumor tissue was obtained for immune microenvironment testing.

### 2.17. Statistical Analysis

The shown data were expressed as mean ± standard deviation (Stdv). Statistics were analyzed with GraphPad Prism (V6.0, GRAPHPAD SOFTWARE. LLC, Santiago, CA, USA)). Student’s *t*-tests were used to compare the means of the different groups. A *p*-value < 0.05 was considered statistically significant [17].

## 3. Results

### 3.1. TAX Inhibits the Proliferation of Lung Cancer Cells

**Figure 1:**
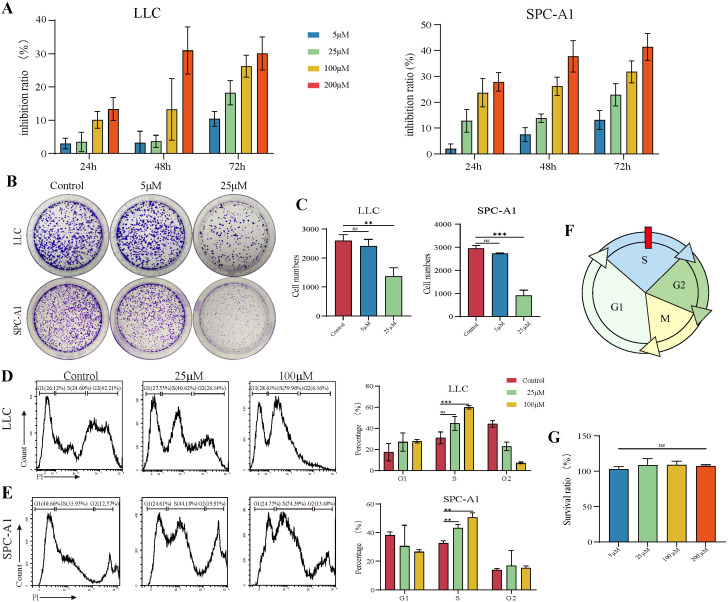
TAX inhibits tumor cell proliferation, arrests the cell cycle in the S-phase, and does not inhibit bronchial epithelial-like cell proliferation. (**A**) CCK-8 assay was carried out and the absorbance was measured at 450 nm, and cell viability calculated. (**B**,**C**) Crystal violet staining was performed to demonstrate the colony-formation ability, and cell counting was performed using Image J software. (**D**,**E**) The percentage of cells in each phase was obtained and statistically analyzed by flow cytometry using PI staining to label the DNA. (**F**) The cell cycle was arrested in the S-phase. (**G**) The viability of HBE cells treated with TAX was measured using the CCK-8 assay. ns, not significant, ** *p* < 0.01, *** *p* < 0.001. (Student’s *t*-test). Data are presented as mean ± stdv.

To evaluate the effects of TAX on lung cancer cells, Lewis lung cancer (LLC) and lung carcinoma SPC-A1 cell lines were treated with different concentrations of TAX for 24 h, 48 h, or 72 h, then cell viability was detected by CCK8 assay. The data showed that TAX inhibited the growth of LLC and SPC-A1 cells in a time- and dose-dependent manner [17] (Figure 1A). The colony-formation assay also demonstrated that TAX inhibited the growth of LLC and SPC-A1 cells (Figure 1B,C). The cell cycle represents a series of integrated events that regulate cell growth, which is important for cancer cell proliferation. Here, we hypothesized that TAX displays inhibitory effects on cell proliferation and colony formation might be achieved by modulating the cell cycle. To this end, LLC and SPC-A1 cells were treated with TAX, and the effect on cell cycles was detected by flow cytometry. It showed that TAX was able to arrest the division of LLC and SPC-A1 cells in the S-phase, thus inhibiting the growth of the cell lines (Figure 1D–F). Meanwhile, to identify whether TAX displayed any effect on normal human cells, we treated human bronchial epithelial cells with TAX, and the result showed that TAX did not inhibit the proliferation of human bronchial epithelial cells (Figure 1G). Taken together, the data indicated that TAX efficiently inhibited the proliferation of lung cancer cells via modulating the division cycle in the S-phase without affecting the activity of normal lung epithelial cells.

### 3.2. TAX Inhibits Lung Cancer Cell Migration by Interfering with EMT

**Figure 2:**
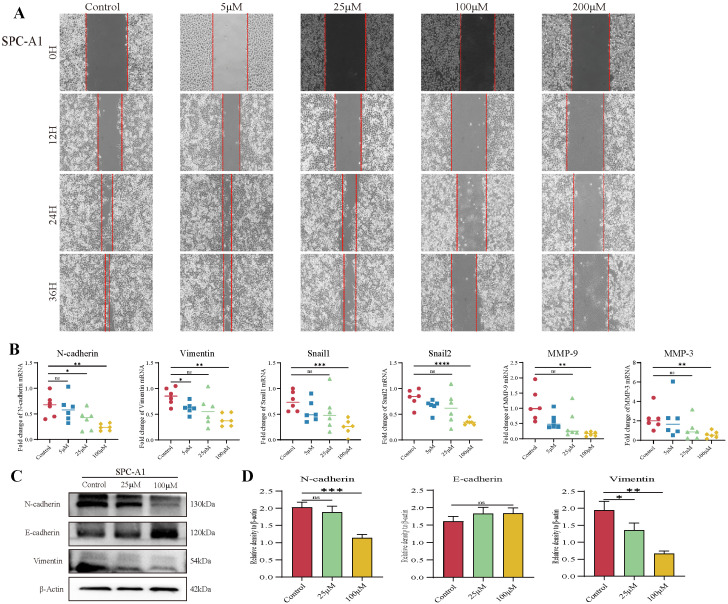
TAX inhibits migration of SPC-A1 cells. (**A**) Representative results of the wound-healing assay with SPC-A1 cells. (**B**) The expression of several key regulators of cell metastasis, adhesion, and migration in SPC-A1 cells was detected by RT-qPCR, after the treatment of TAX for 48 h. (**C**,**D**) The expression of several key EMT-related proteins was detected by western blotting after the treatment of TAX for 48 h. Images were quantified and statistically analyzed. ns, not significant, * *p* < 0.05, ** *p* < 0.01, *** *p* < 0.001, **** *p* < 0.0001. (Student’s *t*-test). Data are presented as mean ± stdv.

Cancer cell migration has been regarded as an integral process in early cancer ontogeny. To measure the effect of TAX on the migration ability of tumor cells, a wound-healing assay was applied. The data demonstrated that TAX can efficiently inhibit the migration of SPC-A1 and LLC tumor cells in a time- and dose-dependent manner (Figure 2A and Figure S1A). In addition, epithelial-mesenchymal transition (EMT) is a process by which cells lose their epithelial properties and gain mesenchymal properties, meaning that tumor cells gain a greater ability to invade and detach. EMT plays a key role in cancer progression, metastasis, and drug resistance [25]. To examine whether TAX influences EMT, the expression of EMT-related key molecules including N-cadherin, Vimentin, Snail, MMP, and E-cadherin were measured in SPC-A1 and LLC tumor cells by RT-qPCR and western-blot after co-culture with TAX. It showed that TAX downregulated the expression of mesenchymal markers N-cadherin, vimentin, snail1, snail2, MMP9 and upregulated the expression of the epithelial marker E-cadherin in a dose-dependent manner in mRNA and protein levels (Figure 2B–D), suggesting that TAX efficiently inhibited the EMT process, thereby inhibiting the migration of lung cancer cells. The data revealed that TAX possesses significant potential to inhibit the migration of lung cancer cells.

### 3.3. Apoptosis Was Predicted as the Main Pathway of TAX in NSCLC by Network Pharmacology

**Figure 3:**
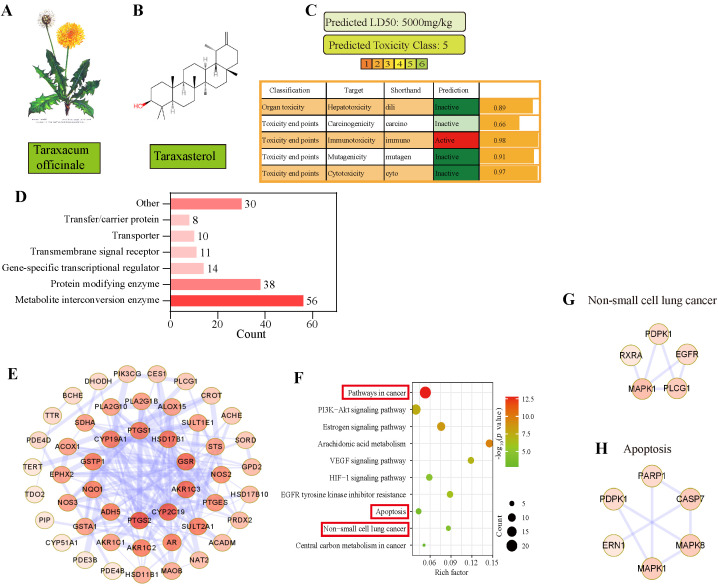
Predicting the targets and safety evaluation of TAX by network pharmacology. (**A**) The image of Taraxacum officinale. (**B**) Using the online structural editor InDraw according to smile number (http://indrawforweb.integle.com/ (accessed on 1 May 2021)) Mapping the 2D chemical structure of TAX. (**C**) Using the ProTox-II Database (https://tox-new.charite.de/protox_II/ (accessed on 1 May 2021)), predicting the drug safety of TAX. (**D**) Acquisition of the targets of action of TAX. (**E**) Construction of a protein-protein interaction (PPI) network. (**F**) KEGG enrichment analysis. (**G**,**H**) construction of a protein-protein interaction (PPI) network involved in non-small cell lung cancer (hsa05223) versus apoptosis (hsa04210).

TAX is one of the main components of *Taraxacum officials* (Figure 3A). To better evaluate the potential effects on NSCLC progression, we first mapped the 2D structure of TAX according to their smile number (Smile C=C1[C@@H](C)[C@@H]2[C@@H]3[C@](CC[C@@]2(C)CC1)(C)[C@@]4(C)[C@@H](CC3)[C@@]5(C)[C@@H](CC4)C(C)(C)[C@@H](O)CC5) using the online structural editor InDraw (http://indrawforweb.integle.com/ (accessed on 1 May 2021)) (Figure 3B) and analyzed whether it was a potential drug for clinical use according to the Lipinski’s “Rule of Five” and the chemical structure. The data showed that TAX harbors a molecular weight of less than 500 KD and a reasonable number of hydrogen bond donors and acceptors, suggesting a promising drug candidate. The drug similarity of TAX is 0.4, and it indicates that the pharmacological activity of TAX is good. In addition, the toxicity of a drug is also critical for preclinical studies; therefore, the ProTox-II Database (https://tox-new.charite.de/protox_II/ (accessed on 1 May 2021)) was used to predict the oral acute toxicity LD50 of TAX compared with other toxicities. The LD50 of TAX is 5000 mg/kg, which means that TAX has almost no hepatotoxicity, carcinogenicity, or teratogenicity in theory (Figure 3C).

Next, the possible pathways and targets of TAX were evaluated by using the Traditional Chinese Medicine System Pharmacology Database and Analysis Platform (https://old.tcmsp-e.com/tcmsp.php (accessed on 1 May 2021)). We found that, among 189 action targets, metabolism-related interaction was strongly involved after TAX treatment (Figure 3D,E). Then, we used a string database (https://string-db.org/ (accessed on 1 May 2021)) to construct the protein interaction network and made the KEGG map for enrichment analysis, and found that the above targets were involved in several pathways, most of which closely correlated to cancer and apoptosis (Figure 3F). Finally, we further constructed the protein interaction network between TAX and NSCLC, and the apoptosis-related proteins; and a series of proteins were screened out (Figure 3G,H). These data indicated that TAX was a potential drug and displayed its antitumor effect by inducing apoptosis in NSCLC.

### 3.4. TAX Induces Early Apoptosis and Alters Mitochondrial Membrane Potential

**Figure 4:**
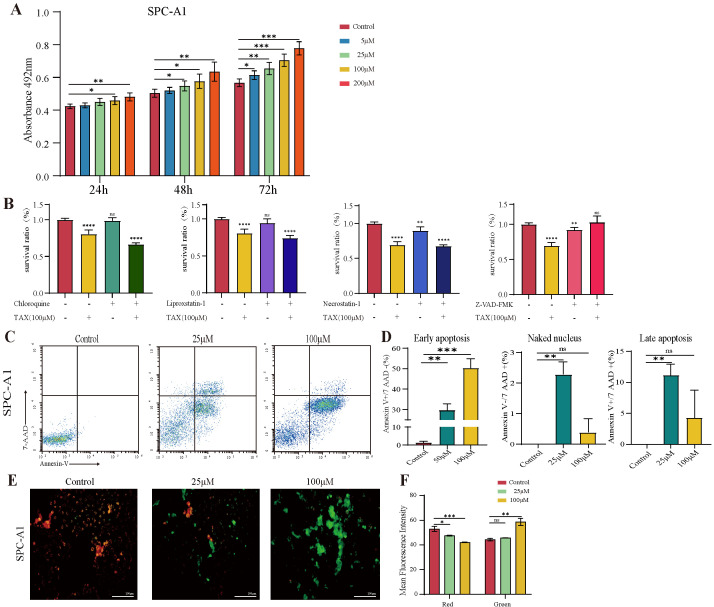
TAX induces the apoptosis of lung cancer cells: (**A**) After SPC-A1 cells were treated with different concentrations of TAX for indicated time periods, LDH release assay was carried out and the absorbance was measured at 492 nm. After SPC-A1 cells were treated with different concentrations of TAX for indicated time periods, LDH release assay was carried out and the absorbance was measured at 492 nm. (**B**) TAX was treated with chloroquine (CQ 25 μM, a potent inhibitor of autophagy), Liproxstatin-1 (Lip 8 μM, a potent ferroptosis inhibitor), necrostatin-1 (Nec-1 10 μM, a potent inhibitor of necroptosis) and Z-VAD-FMK (10 μM, a pan-caspase inhibitor). CCK-8 assay was carried out and the absorbance was measured at 450 nm, and inhibition ratio was calculated. (**C**,**D**) SPC-A1 cells were double-stained with FITC annexin V/7-AAD fluorescence after the treatment of TAX for 48 h to detect early apoptosis, and the percentage of cells in early or late apoptosis was obtained and statistically analyzed. (**E**,**F**) The mitochondrial membrane potential alteration in SPC-A1 cells treated with TAX for 48 h was detected using fluorescence microscopy, and the shift of JC-1 dye from red-to-green fluorescence was a signal of early apoptosis, and the images were quantified and statistically analyzed. Scale bar: 200μm. ns, not significant, * *p* < 0.05, ** *p* < 0.01, *** *p* < 0.001, **** *p* < 0.0001. (Student’s *t*-test). Data are presented as mean ± stdv.

The network pharmacology prediction showed that the potential target of TAX was enriched in apoptosis-related pathways. To further verify this, we performed a series of experiments in vitro. When cells undergo apoptosis or necrosis, the structure of the cell membrane disrupts, resulting in the release of intracellular lactate dehydrogenase (LDH). Therefore, LDH release is considered an important indicator of cell membrane integrity and widely used in cytotoxicity assays. Our data revealed that TAX significantly promoted the release of LDH from LLC and SPC-A1 cells in a time- and dose-dependent manner (Figure 4A and Figure S2A). To further explore the mechanism of TAX-induced cell death, we used the inhibitors of chloroquine, Liproxstatin-1, necrostatin-1, or Z-VAD-FMK in the culture system. These compounds are known to inhibit autophagy, ferroptosis, necroptosis, and caspase, respectively. We observed that only Z-VAD-FMK rescued TAX-induced cell death, indicating that apoptosis rather than other types of cell deaths contributed to TAX-induced cell death (Figure 4B and Figure S2B). Next, an Annexin V/7-AAD dual staining was applied to detect apoptotic cells by flow cytometry. As a result, a significant increase in both the early apoptotic and late apoptotic cells was observed in LLC and SPC-A1 cells after TAX administration (Figure 4C,D and Figure S2C,D). These data indicated that TAX displayed its antitumor effect by inducing tumor cell apoptosis. The process of apoptosis is usually accompanied by the disruption of the mitochondrial transmembrane potential (MTP), which is regarded as one of the earliest events during apoptosis. Therefore, we asked whether TAX could influence MTP. To this end, the JC-1 fluorescence system was introduced, in which the transition of JC-1 dyes from red-to-green fluorescence indicates the decrease of mitochondrial membrane potential, predicting early apoptosis of cells. The results showed that the SPC-A1 cells turned from red to green after administration of TAX, suggesting that TAX treatment did induce the early apoptosis in NSCLC cells (Figure 4E,F).

**Figure 5:**
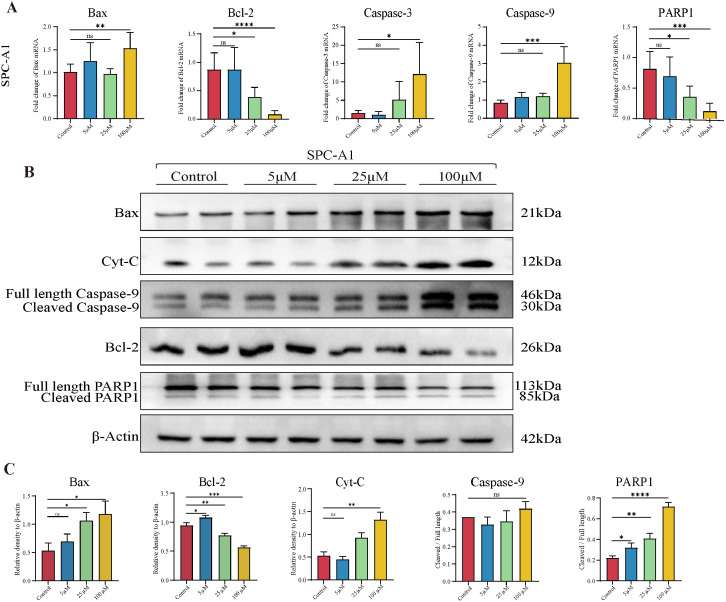
TAX regulates the key apoptosis regulators of SPC-A1 (**A**) The expression of several key apoptosis regulators was detected using RT-qPCR after the treatment of TAX for 48 h in SPC-A1 cells. (**B**,**C**) The expression of several key apoptosis regulatory proteins in SPC-A1 cells was detected using western blotting after the treatment of TAX for 48 h. Images were quantified and statistically analyzed. Ns, not significant, * *p* < 0.05, ** *p* < 0.01, *** *p* < 0.001, **** *p* < 0.0001. (Student’s *t*-test). Data are presented as mean ± stdv.

Next, we also examined a series of apoptosis-related molecules at the mRNA level by RT-qPCR. The data demonstrated that TAX promoted the expression of the pro-apoptotic gene Bax, Caspase-3, and Caspase-9, and inhibited the expression of the anti-apoptotic gene Bcl-2 and oncogene Poly ADP ribose polymerase (PARP1) in SPC-A1 and LLC tumor cells (Figure 5A and Figure S3A). Next, the protein levels of these genes were also investigated, and the expression of Bax, Caspase-9, Bcl-2, and PARP1 showed a similar tendency to mRNA levels. In addition, we also detected the elevated protein levels of Cytochrome C (Cyt-C), which plays an important role in endogenous apoptosis (Figure 5B,C and Figure S3B,C).

Taken together, these data demonstrated that TAX-induced early apoptosis, as indicated by JC-1 fluorescence changes, altered related genes and proteins in lung cancer cells.

Increasing evidence has shown that ferroptosis and autophagy are also intimately associated with cancer initiation, progression, and suppression. To evaluate whether TAX also displayed any modulatory effects on ferroptosis and autophagy, first, we examined the release of reactive oxygen species (ROS), an indicator associated with lipid peroxidation during ferroptosis by flow cytometry, and detected the mRNA expression of GPX4, SLC7a11, and Nrf2, which are correlated with glycine metabolism and iron transport in the presence or absence of TAX. Interestingly, the results showed that TAX did not alter the levels of ROS, GPX4, or SLC7a11 (Figure S4A,B). Next, we examined the mRNA levels of LC3-II and the key molecules in the autophagy pathway, and found that TAX also did not influence their expression at the transcriptional level (Figure S4C). Some mRNA expressions increased, such as Nrf2 or P62, but the western blot assay failed. We considered that special situations such as post-transcriptional translation may arise. Therefore, we conclude that TAX exerts its anti-tumor effect mainly through the apoptosis pathway, rather than ferroptosis or autophagy.

### 3.5. The Therapeutic Effects of TAX on NSCLC Growth in the Subcutaneous Tumor Model

**Figure 6:**
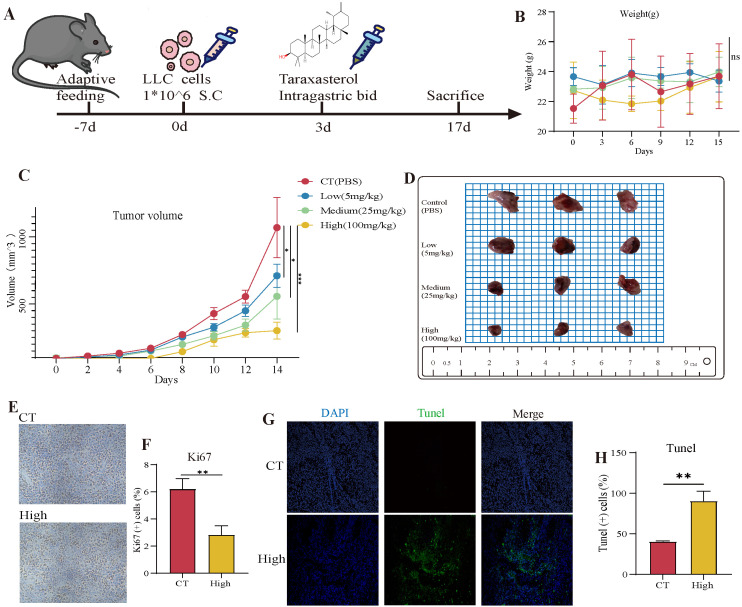
TAX exerts antitumor effects in vivo: (**A**) Subcutaneous tumor inoculation and administration schedule in mice. (**B**, **C**) Body weight and tumor volume in mice. (**D**) Tumor image in mice. (**E**, **F**) Immunohistochemistry of ki67 staining and statistical analysis of tumor tissue. (**G**, **H**) TUNEL fluorescence detection and statistical analysis of tumor tissue. ns, not significant, * *p* < 0.05, ** *p* < 0.01, *** *p* < 0.001. (Student’s *t*-test). Data are presented as mean ± stdv.

To evaluate the therapeutic potential of TAX in vivo, we generated a subcutaneous xenograft tumor mouse model. First, LLC cells were subcutaneously injected into C57BL/6 mice. When the tumors grew to around 100 mm^3^ in size, the mice were randomly divided into different experimental groups and administrated with PBS or different concentrations of TAX (low concentration: 5 mg/kg, medium concentration: 25 mg/kg; high concentration: 100 mg/kg) (Figure 6A). Results showed that there was no significant difference in body weight among these experimental groups (Figure 6B). Importantly, the growth of LLC-induced xenografts was significantly inhibited after TAX treatment, as verified by the decreased tumor volume and weight compared with those in the control group, and the inhibition effect of TAX showed a dose-dependent manner (Figure 6C,D). In addition, we also found that TAX administration significantly inhibited tumor cells as indicated by reduced Ki67+ cells (Figure 6E,F) and enhanced positive Tunel staining (Figure 6G,H) compared with the control group, indicating that TAX inhibited tumor growth mainly by inhibiting proliferation and inducing apoptosis of tumor cells.

**Figure 7:**
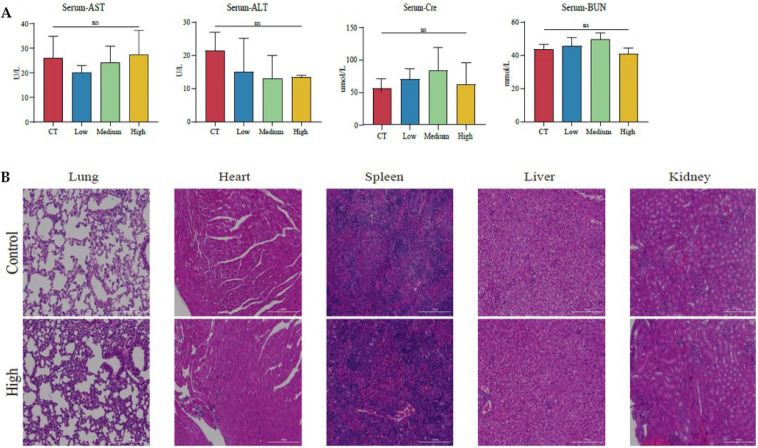
The use of TAX has no obvious side effects: (**A**) Serum ALT, AST, BUN, and Cre levels were not changed after TAX treatment. (**B**) H&E staining of major organs after 2 weeks of TAX administration. Scale bar: 200μm. ns, not significant. (Student’s *t*-test). Data are presented as mean ± stdv.

Furthermore, we evaluated the possible side-effects caused by TAX in vivo. The enzymes reflecting liver and kidney function, and the morphological alterations of vital organs were examined. It showed that TAX neither affected the serum biochemical biomarkers of liver and kidney including aspartate aminotransferase (AST), alanine aminotransferase (ALT), creatinine (Cre), or blood urea nitrogen (BUN), nor altered the morphology of the heart, spleen, liver, or kidney as detected by H&E staining (Figure 7A,B), suggesting that TAX treatment did not cause detectable toxicity in mice. This data is consistent with the safety assessment performed by preliminary bioinformatics, as well as the results of the cellular experiments (Figure 1F and Figure 3C).

### 3.6. TAX Inhibits EMT, Induces Tumor Cell Apoptosis, and Modulates the Internal Immune Microenvironment In Vivo

**Figure 8:**
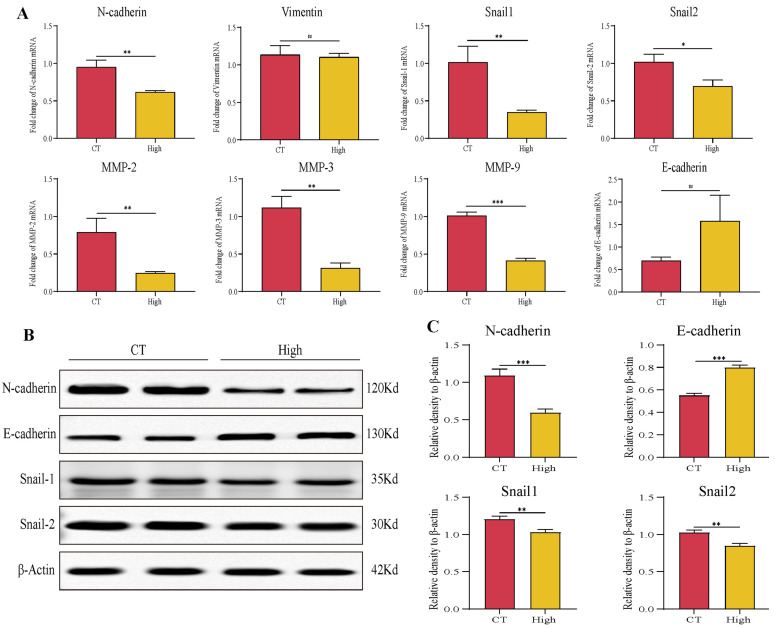
TAX inhibits the EMT in vivo: (**A**) The expression of several key EMT regulators was detected using RT-qPCR after the treatment of TAX in tumor tissue of tumor-bearing mice. (**B**,**C**) The expression of several key EMT regulators was detected using western blotting after the treatment of TAX in tumor tissue of tumor-bearing mice. Images were quantified and statistically analyzed. ns, not significant, * *p* < 0.05, ** *p* < 0.01, *** *p* < 0.001. (Student’s *t*-test). Data are presented as mean ± stdv.

**Figure 9:**
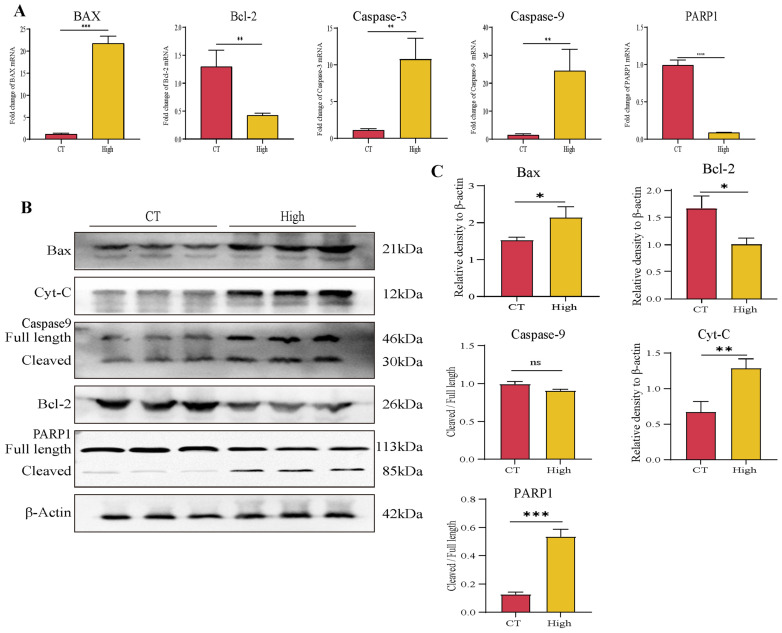
TAX induces apoptosis in vivo: (**A**) The expression of several key apoptosis regulators was detected using RT-qPCR after the treatment of TAX in tumor tissue of tumor-bearing mice. (**B**,**C**) The expression of several key apoptosis regulators was detected using western blotting after the treatment of TAX in tumor tissue of tumor-bearing mice. Images were quantified and statistically analyzed. ns, not significant, * *p* < 0.05, ** *p* < 0.01, *** *p* < 0.001. (Student’s *t*-test). Data are presented as mean ± stdv.

To further analyze whether in vivo administration of TAX indeed inhibited EMT and induced apoptosis, we detected a series of molecules related to EMT including N-cadherin, E-cadherin, Snail1, Snail2, MMP-2/3/9 and apoptosis-related markers such as Bax, Caspase-9, Bcl-2, Cyt-c and PARP1 in tumor by RT-qPCR and western blot. It showed that TAX downregulated the expression of mesenchymal markers N-cadherin, snail1, snail2, MMP2, MMP3, MMP9 and upregulated the expression of the epithelial marker E-cadherin in protein or RNA levels, suggesting that TAX efficiently inhibited the EMT process in vivo (Figure 8A–C). Next, we investigated the effect of TAX on apoptosis in the tumor in vivo. TAX promoted the expression of the pro-apoptotic molecules such as Bax, Caspase-3, Caspase-9, Cyt-C and inhibited the expression of the anti-apoptosis molecules such as Bcl-2 and PARP1, indicating that TAX efficiently induced apoptosis in tumor cells in mRNA or protein (Figure 9A–C) levels. These results are consistent with our previous cellular assays in vitro, further proving that TAX effectively inhibited tumor progression by blocking the EMT and inducing apoptosis in tumor cells.

**Figure 10:**
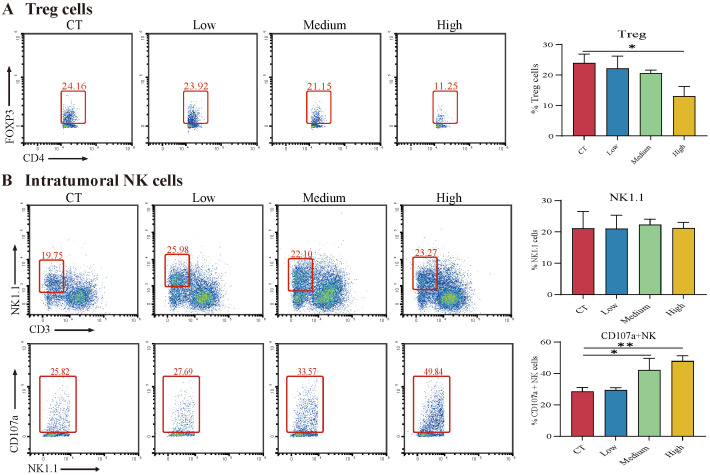
TAX modulates immune cells in the tumor microenvironment in vivo: (**A**,**B**) Changes of Treg cells and NK cells in the tumor microenvironment. ns, not significant, * *p* < 0.05, ** *p* < 0.01. (Student’s *t*-test). Data are presented as mean ± stdv.

With the aim to explore the alteration of the tumor microenvironment in mice after TAX treatment, the immune cells in TME, including T cells, Treg cells, macrophages, and NK cells were examined by flow cytometry. Infiltration of Treg cells in tumors correlates with poor prognosis [26]. Here, we found that high doses of TAX significantly reduced the proportion of Treg cells (Figure 10A). NK cells kill target cells by secreting lysosomal granules, which leads to the expression of CD107a on the membrane of NK cells [27]. Therefore, the expression of CD107a was regarded as a marker of NK cell activation and toxic degranulation. The data showed that TAX dramatically upregulated the proportion of CD107a+ NK cells, although there is no significant difference in the frequency of NK cells (Figure 10B). Furthermore, no significant differences were detected for Th1, Th17, IFNγ+ CD8 T cells, M1 and M2 macrophages, or perforin+ NK cells (Figure S5A–C). The data revealed that regulation of TME by TAX in vivo might be another possible mechanism of its anti-tumor effect.

## 4. Discussion

TAX has the ability to suppress the growth of colorectal cancer [16], melanoma [14], and papillary thyroid cancer [15]. However, the effects of TAX on lung cancer are still not known. In this study, we studied the role and potential mechanism of TAX in restraining NSCLC both in vivo and in vitro. Our data demonstrated that TAX inhibited the proliferation and migration of LLC and SPC-A1 cell lines in a time- and dose-dependent manner. Further study revealed that TAX-mediated antitumor effects were achieved mainly by inducing tumor cell apoptosis and modulating TME.

Accumulating evidence shows that TAX exerts antitumor effects through several signaling pathways including Wnt/β-catenin, PI3K/Akt. One study found that TAX promoted oncogenic gene RNF31 degradation by activating autophagy, and the depletion of RNF31 alleviated cancer suppressor gene p53 degradation [16]. It is widely known that targeting p53, including restoring its normal function or degradation, can trigger tumor cell death and elimination [28]. Although the inhibitory effect of TAX in tumor growth is well described, its pharmacological mechanisms are obscure. Therefore, exploring the novel mechanisms for its antitumor effects may pave the way for the clinical application of TAX.

Apoptosis is one kind of programmed cell death characterized by a series of morphological changes in the structure of the cells, including phosphatidylserine efflux from the inner leaflet of plasma membrane lipids and potential mitochondrial membrane alteration [29]. High expression of Bax and low expression of Bcl-2 increase the permeability of the mitochondrial permeability of the outer membrane, leading to the release of cytochrome C into the cytoplasm. The released cytochrome C activates caspase-9, which in turn activates caspase-3. Activated caspase-3 cleaves PARP1; both activations of caspase-3 and PARP1 were accepted as a hallmark of apoptosis [30]. PARP1 is essential for DNA repair and maintaining genomic integrity. Olaparib, the inhibitor of PARP1, has become the first-line standard treatment for advanced ovarian cancer patients in clinics [31]. In our study, we observed that phosphatidylserine was flipped from the inside to the outside of the cell membrane and the mitochondrial membrane potential was decreased after TAX treatment. In addition, TAX decreased Bcl-2 and increased Bax and Cytochrome C levels, indicating that TAX efficiently induced NSCLC cell apoptosis. Importantly, the mitochondria-mediated pathway was identified as the intracellular potential action target upon modulation by TAX. In addition, TAX also upregulated the expression of caspase-9 and PARP1, further proving that the underlying mechanism of TAX-mediated inhibition of NSCLC progression is due to the induction of apoptosis in NSCLC cells via the apoptotic pathway.

Targeting TME is believed to be an emerging standard treatment leading to a new era of cancer therapy [32]. One study performed a single-cell sequencing of clinical samples from NSCLC patients and found that NSCLC patients suffered from long-term chronic antigenic stimulation, and had T-cell exhaustion and massive Treg cell infiltration, followed by expression of a series of cell surface suppressor molecules. This led to the suppression of T-cell function and immune escape of tumor cells [33,34]. T lymphocytes, especially CD4+ T cells and CD8+ T cells, play an extremely important role in TME [35]. CD4+ T cells attack tumor cells in multiple ways, and CD8+ T cells release a variety of cytokines to clear tumor cells upon recognition of tumor-specific antigens via T-cell receptors. Treg cells suppress antitumor immunity and lead to poor prognosis in NSCLC patients [36]. NK cells directly recognize and kill tumor cells by secreting lysosomal granules, which leads to the expression of CD107a on the membrane of NK cells [27,37]. CD107a was found to be a marker of NK cell activation and toxic degranulation [38]. Several drugs targeting various components of TME have been approved for clinical use in the past decade. The drugs or molecule compounds that reverse the immune suppression environment including depleting Treg cells or attenuating their inhibitory activity, in combination with other immunotherapies, would make current cancer immunotherapies more effective in clinical practice [39]. Our results show that TAX reduced Treg infiltration in TME, which provides evidence that TAX may serve as an alternative option as an immunotherapy to improve clinical efficacy for NSCLC treatment. As critical as T-cells, NK cells also play a tremendous role in antitumor immunity. The mechanism of NK cell-mediated anti-tumor function is mainly reliant on degranulation, which results in the lysis of tumor cells. It reported that surface expression of CD107a is highly correlated with NK cell cytotoxic activity [27,38]. Our results showed that TAX significantly increased the proportion of CD107a+ NK cells in in vivo tumor models, indicating that TAX likely promoted NK cell-mediated degranulation and enhanced the killing function of NK cells. However, current data about the effects of TAX on TME is quite preliminary and worthy of further study.

## 5. Conclusions

**Figure 11:**
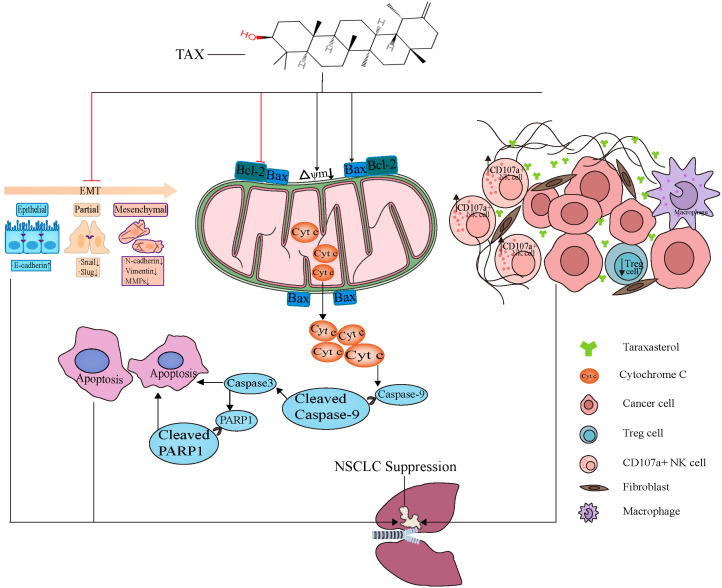
Schematic depiction of the anti-NSCLC effects of TAX.

In summary, our data demonstrated that TAX exerts its antitumor effects by inhibiting EMT, modulating the tumor microenvironment, and promoting tumor cell apoptosis (Figure 11). This work provides solid evidence that TAX may serve as a promising drug candidate for NSCLC treatment. Of course, the study of the function and mechanism of TAX-mediated inhibitory effects on NSCLC is still superficial. Therefore, the precise mechanism and therapeutic efficacy of TAX on NSCLC cells need to be further investigated in the future.

## Figures and Tables

**Table 1:** Primer sequence.

Species	Gene	Primer Sequence (5′-3′)
Human	β-actin-F	TCGTGCGTGACATTAAGGAG
	β-actin-R	ATGCCAGGGTACATGGTGGT
Human	PARP1-F	CCAAGCCAGTTCAGGACCTCAT
	PARP1-R	GGATCTGCCTTTTGCTCAGCTTC
Human	Bax-F	CGTGAAGGACGCACGTTC
	Bax-R	AGTGCTTGGAGATCGCACAG
Human	Bcl-2-F	AGGCGGCAGTTAAAGCTCAT
	Bcl-2-R	CGCATCTGCGGCAAGAG
Human	Caspase-3-F	TCACCATGGCTCAGAAGCAC
	Caspase-3-R	CAACTTCCTAAAATGGTTTGAGATGTGTT
Human	Caspase-9-F	GAGGACGAGCCCTTCTGATG
	Caspase-9-R	ATGACAAGAGGTGTTTGTTTCTGCT
Human	N-Cadherin-F	CCTCCAGAGTTTACTGCCATGAC
	N-Cadherin-R	GTAGGATCTCCGCCACTGATTC
Human	E-Cadherin-F	GCCTCCTGAAAAGAGAGTGGAAG
	E-Cadherin-R	TGGCAGTGTCTCTCCAAATCCG
Human	Vimentin-F	AGGCAAAGCAGGAGTCCACTGA
	Vimentin-R	ATCTGGCGTTCCAGGGACTCAT
Human	Snail-1-F	TGCCCTCAAGATGCACATCCGA
	Snail-1-R	GGGACAGGAGAAGGGCTTCTC
Human	Snail-2-F	ATCTGCGGCAAGGCGTTTTCCA
	Snail-2-R	GAGCCCTCAGATTTGACCTGTC
Human	MMP3-F	CACTCACAGACCTGACTCGGTT
	MMP3-R	AAGCAGGATCACAGTTGGCTGG
Human	MMP9-F	GCCACTACTGTGCCTTTGAGTC
	MMP9-R	CCCTCAGAGAATCGCCAGTACT
Human	SLC7a11-F	TCCTGCTTTGGCTCCATGAACG
	SLC7a11-R	AGAGGAGTGTGCTTGCGGACAT
Human	GPX4-F	ACAAGAACGGCTGCGTGGTGAA
	GPX4-R	GCCACACACTTGTGGAGCTAGA
Human	LC3II-F	GAGAAGCAGCTTCCTGTTCTGG
	LC3II-R	GTGTCCGTTCACCAACAGGAAG
Human	P62-F	TGTGTAGCGTCTGCGAGGGAAA
	P62-R	AGTGTCCGTGTTTCACCTTCCG
Mouse	PARP1-F	CTCTCCCAGAACAAGGACGAAG
	PARP1-R	CCGCTTTCACTTCCTCCATCTTC
Mouse	Bax-F	AGGATGCGTCCACCAAGAAGCT
	Bax-R	TCCGTGTCCACGTCAGCAATCA
Mouse	Bcl-2-F	CCTGTGGATGACTGAGTACCTG
	Bcl-2-R	AGCCAGGAGAAATCAAACAGAGG
Mouse	Caspase-3-F	GGAGTCTGACTGGAAAGCCGAA
	Caspase-3-R	CTTCTGGCAAGCCATCTCCTCA
Mouse	Caspase-9-F	GCTGTGTCAAGTTTGCCTACCC
	Caspase-9-R	CCAGAATGCCATCCAAGGTCTC

## Data Availability

The original contributions presented in the study are included in the article. Further inquiries can be directed to the corresponding authors.

## Supplementary Materials

The following supporting information can be downloaded at: https://www.mdpi.com/article/10.3390/cancers14194645/s1, Figure S1: TAX inhibits the migration of lung cancers. (a) LLC cell migration was detected by wound-healing assay; Figure S2. TAX induces the apoptosis of Lewis lung cancer cells; Figure S3. TAX regulates the key apoptosis regulators of LLC; Figure S4. The anti-tumor effects of TAX on SPC-A1 cells might have no connection with ferroptosis and autophagy; Figure S5. TAX modulates immune cells in the tumor microenvironment in vivo.
