# Supplementary material for: Taraxasterol Inhibits Tumor Growth by Inducing Apoptosis and Modulating the Tumor Microenvironment in Non-Small Cell Lung Cancer
Source: Cancers (Basel). 2022 Sep 24;14(19):4645. doi: 10.3390/cancers14194645 (PMC9562636; doi:10.3390/cancers14194645)
Supplement: Supplementary file 1 [file cancers-14-04645-s001.zip › cancers-1921606-supplementary File S1.pdf]

CCK-8 assay

Cell Counting Kit-8 (CCK-8) assay is a sensitive colorimetric method to determine the number of living cells in the proliferation and cytotoxicity tests. The detection sensitivity is higher than the traditional MTT or MTS method.

Here are the steps to use：

1.The seed cells were placed in 96 well plates at a density of 1×10^3-1×10^4 cells/well in 100 μ L medium. The cells were incubated in a CO2 incubator at 37 ℃ for 24 hours.

2.Add substances of different concentrations to the plate.

3.Incubate the culture plate in the incubator for an appropriate time (for example, 6, 12, 24 or 48 hours).

4.Add 10 to each hole of the plate with a repeated pipette μ LCCK8 solution. Be careful not to introduce bubbles into the hole, as they will interfere with O.D. readings.

5.Incubate the culture plate in the incubator for 1-4 hours.

6.Before reading the orifice plate, it is important to mix gently on the track vibrator for 1 minute to ensure uniform color distribution.

7.Use the microplate reader to measure the absorbance at 450 nm.

LDH release assay

LDH Cytotoxicity Assay Kit can detect the activity of lactate dehydrogenase released during cytotoxicity. The basic principle is that under the action of lactate dehydrogenase, NAD+ is reduced to produce NADH, NADH and INT (2-p-iodophenyl-3-nitrophenyl tetrazolium chloride) are catalyzed by lipoamide dehydrogenase to produce NAD+ and strong chromogenic substance formazan, and an absorption peak is generated under the wavelength of 490nm, so that the activity of lactate dehydrogenase can be quantified by colorimetry. The absorbance was linear positive correlation with LDH activity.

Here are the steps to use：

1.60 for each hole μ L LDH detection working fluid.

2.Evenly mix and incubate in dark at room temperature (about 25 º C) for 30min (wrapped with aluminum foil and then placed on a horizontal or side sway shaker to slowly shake). Then measure the light absorption at 490nm Degrees.

Cell cycle assay

Cell Cycle Staining Kit.

The basic principle is that Propidium iodide (PI) is a fluorescent dye of double stranded DNA. When propidium iodide is combined with double stranded DNA, it can produce fluorescence, and the fluorescence intensity is proportional to the content of double stranded DNA. After the DNA in cells is stained with propidium iodide, the DNA content of cells can be determined by flow cytometry. According to the distribution of DNA content, the cell cycle can be analyzed.

Here are the steps to use：

1.Collection 2×10^5-1×10^6 cells were centrifuged and the supernatant was discarded. Wash with PBS once, and discard the supernatant by centrifugation.

2.Add 1 ml of DNA Staining solution and 10 μ L Permeabilization solution, vortex oscillation for 5 - 10 seconds, and mixing. Incubation at room temperature and away from light 30 Minutes.

Measurement of the cell apoptosis

The basic principle is that Annexin V, a member of the annexin family of intracellular proteins, binds to phosphatidylserine (PS) in a calcium dependent manner. PS exists in the inner layer of normal cell plasma membrane, but at the early stage of apoptosis, membrane asymmetry is lost, and PS translocates to the cell surface. Fluorescent labeled Annexin V can specifically bind to it, indicating that the cell is an apoptotic cell.

Here are the steps to use：

1.Collect 1×10^6-3×10^6 cells were centrifuged and washed twice with precooled PBS, and the supernatant was discarded.

2.Add 500 μ L Apoptosis Positive Control Solution is suspended again and incubated on ice for 30 minutes.

3.Centrifugally wash with precooled PBS and discard the supernatant.

4.Add proper amount of precooling 1 × The Binding Buffer was resuspended and mixed with the same number of untreated living cells. Add precooling 1 ×

The Binding Buffer is supplemented to 1.5 ml and divided into three equal tubes, one of which is a blank control tube and two of which are single dye tubes.

5.Add 5 respectively to the single dye tube μ L Annexin V-PE or 10 μ L 7-AAD, incubate at room temperature and away from light for 5 minutes.

6.On the flow cytometer, use a blank tube to adjust the voltage of FSC, SSC and fluorescence channel, and use a single dye tube to adjust fluorescence under this voltage condition Compensation of channels.

Measurement of ROS

The ROS Assay Kit is a kit for detecting active oxygen using fluorescent probe DCFH-DA. DCFH-DA itself has no fluorescence and can pass through the cell membrane freely. After entering the cell, it can be hydrolyzed by the esterase in the cell to generate DCFH. DCFH cannot penetrate the cell membrane, which makes the probe easy to be loaded into the cell. Reactive oxygen species in cells can oxidize non fluorescent DCFH to generate fluorescent DCF. The level of reactive oxygen species in cells can be determined by detecting the fluorescence of DCF.

Here are the methods to use：

Dilute DCFH-DA with serum free culture solution according to 1:1000 to make the final concentration 10 μ mol/l. remove Add DCFH-DA with appropriate volume of diluted cell culture medium. The added volume should be sufficient to cover the cells. Generally, one hole of the six-hole plate should be added and diluted DCFH-DA shall not be less than 1ml. Incubate in 37 º C cell incubator for 20 minutes. Wash the cells with serum-free cell culture solution for three times to fully remove the cells that do not enter the cell DCFH-DA. Use 488nm excitation wavelength and 525nm emission wavelength to detect the intensity of fluorescence.

Mitochondrial membrane potential detection

mitochondrial membrane potential assay kit with JC-1 is a fast and sensitive kit for detecting changes in membrane potential of cells, tissues or purified mitochondria with JC-1 as a fluorescent probe, which can be used for early detection of apoptosis. JC-1 is widely used to detect mitochondrial membrane potential Δ Ψ M is an ideal fluorescent probe. It can detect the membrane potential of cells, tissues or purified mitochondria. When the membrane potential of mitochondria is high, JC-1 aggregates in the matrix of mitochondria to form polymers (J-aggregates), which can produce red fluorescence; When the mitochondrial membrane potential is low, JC-1 cannot be gathered in the mitochondrial matrix. At this time, JC-1 is a monomer, which can produce green fluorescence. In this way, it is very convenient to detect the change of mitochondrial membrane potential through the change of fluorescence color. The relative proportion of red green fluorescence is often used to measure the proportion of mitochondrial depolarization. The decrease of mitochondrial membrane potential is a marker event in the early stage of apoptosis. The decline of cell membrane potential can be easily detected by the change of JC-1 from red fluorescence to green fluorescence, and the change of JC-1 from red fluorescence to green fluorescence can also be used as a detection indicator of early apoptosis.

Here are the steps to use：

1.Wash cells with PBS or other appropriate solutions once, and add 1ml of cells for culture liquid. The cell culture medium may contain serum and phenol red.

2.Add 1ml JC-1 dye working solution and fully mix. Incubate in cell incubator at 37 º C for 20 minutes.

3.After incubation at 37 º C, the supernatant was aspirated and washed twice with JC-1 dye buffer.

4.Add 2ml of cell culture medium, which can contain serum and phenol red.

5.Observe under fluorescence microscope.

Wound-healing assay

Wound-healing assay is a simple and convenient method to determine the migration, movement and repair ability of cells. Similar to the wound healing model in vitro, on the

monolayer adherent cells cultured in vitro culture dishes or plates, use a small amount of gun head or other hard objects to draw lines in the central area of cell growth, remove the cells in the central part, and then continue to culture the cells until the time set in the experiment. Take out the cell culture plate, and observe whether the peripheral cells grow to the central scratch area, to judge the growth and migration ability of cells.

Here are the steps to use：

1.Prepare cells, culture medium and culture insert.

2.As shown in the figure, inoculate the cells into the Insert in the middle of Dish, and then remove the Insert with tweezers to generate 500 μ M wide scratches.

3.Take photos every 4-6 hours

Colony-formation assay

Colony-formation assay is an important technical method used to detect the proliferation ability, invasiveness, sensitivity to killing factors and other items of cells.

Here are the steps to use：

1.After digesting the trypsin of cells in logarithmic growth phase, the complete medium (basic medium+10% fetal bovine serum) was resuspended into cell suspension and counted;

2.Cell inoculation: inoculate 400-1000 cells/well in each experimental group in 6-well plate culture plate (generally 700 cells/well according to cell growth);

3.Continue to culture for 14 days or until the number of cells in most single clones is more than 50. Change the medium every 3 days and observe the cell status;

4.After cloning, take pictures of the cells under the microscope, and then wash them with PBS once. Add 1 mL of 4% paraformaldehyde into each well to fix them for 30-60 min, and wash them with PBS once;

5.Add 1ml of crystal violet dye into each well to dye cells for 10-20 minutes;

6.PBS washed cells several times, dried them, and took photos with digital camera

Western Blot Analysis

Western blot is a technology commonly used to isolate and identify proteins in research. It uses SDS polyacrylamide gel electrophoresis (SDS-PAGE) to separate various proteins contained in the specified sample, then transfers the separated proteins to nitrocellulose or PVDF membrane, and then incubates the membrane with specific antibodies to the sensitive target proteins. In the process of membrane washing, the unconjugated antibody is washed away, leaving only the antibody bound to the target protein. Finally, the bound antibody is detected by fluorescence scanning.

Here are the simple steps:

1.Prepare samples;

2.SDS-PAGE polyacrylamide gel electrophoresis;

3.Membrane transfer;

4.Membrane sealing and antibody incubation;

5.development.

mRNA expression Analysis

RT-qPCR is to add fluorescent groups in the PCR reaction, continuously monitor the sequence of fluorescent signals and changes in signal strength, and instantly analyze the initial of the target gene TUNEL fluorescence detection.

Here are the simple steps:

1.mRNA extraction;

2.reverse transcription;

3.amplification;

4.analysis.

Detection of the immune microenvironment in tumor

In the tumor microenvironment, each immune cell has a specific function to identify and remove tumor cells. By marking and typing immune cells with different fluorescent antibodies, and analyzing the proportion of immune cells in different populations, we can distinguish the subtle differences of cell surface markers.

Here are the simple steps:

1.Isolation of tumor samples;

2.Mechanical dissociation of tumor cells;

3.Cleaning and filtering cells;

4.Flow antibody incubation;

5.Computer detection by flow cytometry.

In vivo tumor model

Subcutaneous transplantation tumor model is an important method for evaluating the in vivo efficacy of preclinical drugs, which is widely used in biological research and drug development.

Here are the simple steps:

1.Select suitable tumor forming cells for culture;

2.According to a certain number of cells (1×10^6-10^7 cells) injection;

3.Feeding until tumor body can be seen by naked eye;

4.Measurement of tumor size;

5.The tumor was removed for subsequent treatment.

Statistical analysis

Statistical analysis is an important method to judge whether there is statistical difference between groups.
