# Supplementary material for: Taraxasterol Inhibits Tumor Growth by Inducing Apoptosis and Modulating the Tumor Microenvironment in Non-Small Cell Lung Cancer
Source: Cancers (Basel). 2022 Sep 24;14(19):4645. doi: 10.3390/cancers14194645 (PMC9562636; doi:10.3390/cancers14194645)
Supplement: Supplementary file 1 [file cancers-14-04645-s001.zip › cancers-1921606-supplementary.pdf]

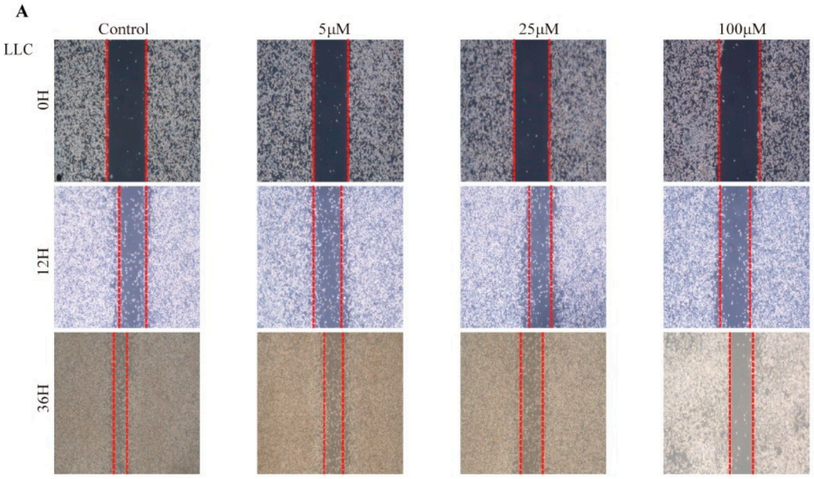


**Figure S1.** TAX inhibits the migration of lung cancers. (**a**) LLC cell migration was detected by wound-healing assay.

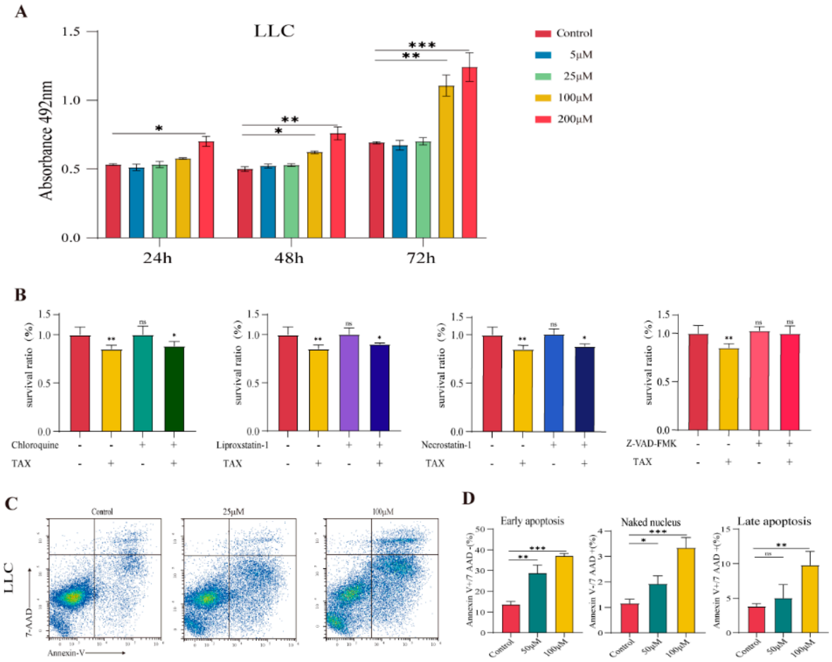


**Figure S2.** TAX induces the apoptosis of Lewis lung cancer cells: (**a**) After LLC cells were treated with different concentrations of TAX for indicated time periods, LDH release assay was carried out and the absorbance was measured at 492 nm. (**b**) TAX was co-cultured with chloroquine (CQ 25μM, a potent inhibitor of autophagy), Liproxstatin-1(Lip 8μM, a potent ferroptosis inhibitor) necrostatin-1 (Nec 10μM, a potent inhibitor of necroptosis) and Z-VAD-FMK (10μM, a pan caspase inhibitor). CCK-8 assay was carried out and the absorbance was measured at 450 nm, and inhibition ratio was calculated. (**c, d**) LLC cells were double-stained with FITC annexin V / 7-AAD fluorescence after the treatment of TAX for 48h to detect early or late apoptosis, and the percentage of cells in early apoptosis was obtained and analyzed statistically. ns, not significant, * $p < 0.05$, **$p < 0.01$, ***$P < 0.001$.

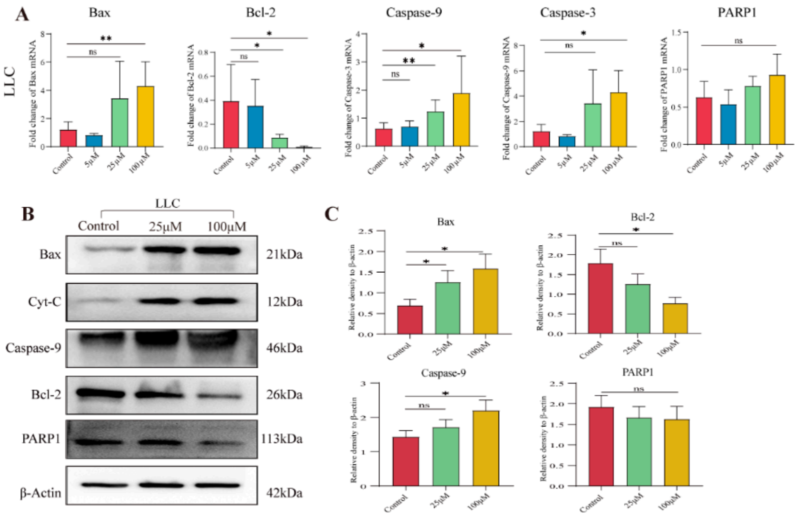


**Figure S3.** TAX regulates the key apoptosis regulators of LLC: (**a**)The expression of several key apoptosis regulators was detected using RT-qPCR after the treatment of TAX for 48 h in LLC cells. (**b, c**) The expression of several key apoptosis regulatory proteins in LLC cells was detected using Western blotting after the treatment of TAX for 48h. Images were quantified and statistically analyzed. ns, not significant, * $p < 0.05$, ** $p < 0.01$, *** $P < 0.001$.

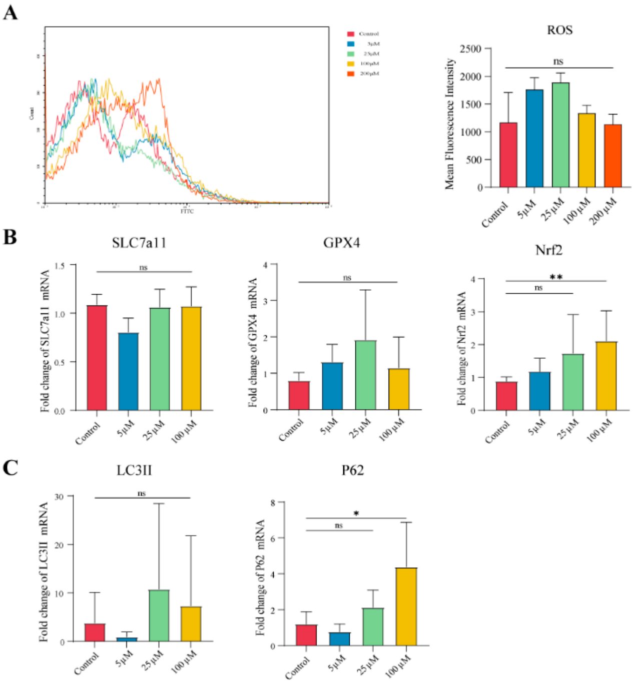


**Figure S4.** The anti-tumor effects of TAX on SPC-A1 cells might have no connection with ferroptosis and autophagy: (**a**) Intracellular ROS levels in SPC-A1 cells were detected using the fluorescent probe DCFH-DA after the treatment of TAX for 48 h, and the mean fluorescence intensity was obtained and analyzed statistically. (**b**) The mRNA expression of several key regulatory factors on the ferroptosis pathway in SPC-A1 cells was detected by RT-qPCR after the treatment of TAX for 48h. (**c**) The mRNA expression of several key regulatory factors on the autophagy pathway in SPC-A1 cells was detected by RT-qPCR after the treatment of TAX for 48h.

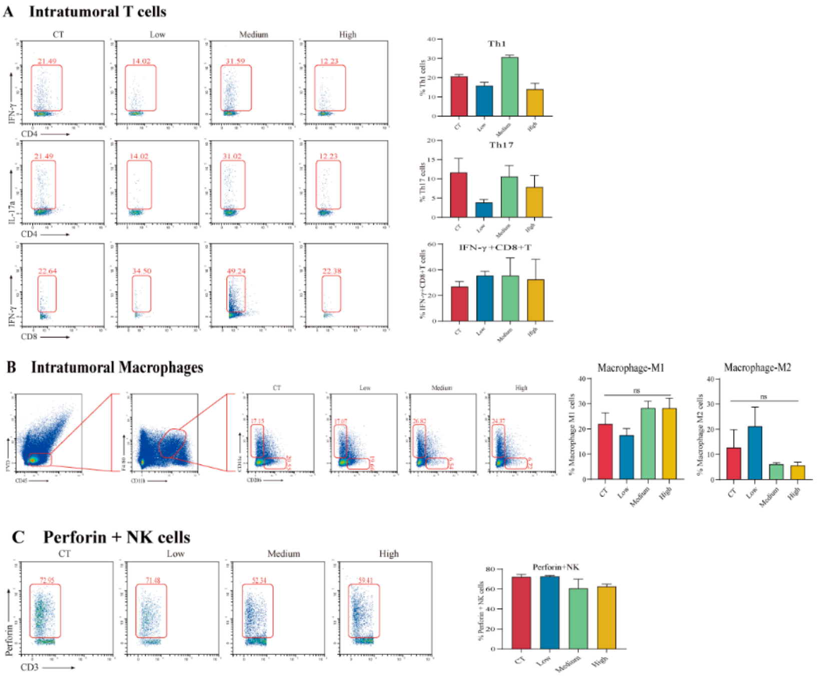

**Figure S5.** TAX modulates immune cells in the tumor microenvironment in vivo: (**a, b, c**) Changes of T cells, macrophages and NK cells in the tumor microenvironment. ns, not significant, * p < 0.05, **p < 0.01, ***P<0.001.
