# Supplementary figures and images for: Taraxasterol Inhibits Tumor Growth by Inducing Apoptosis and Modulating the Tumor Microenvironment in Non-Small Cell Lung Cancer
Source: Cancers (Basel). 2022 Sep 24;14(19):4645. doi: 10.3390/cancers14194645 (PMC9562636; doi:10.3390/cancers14194645)

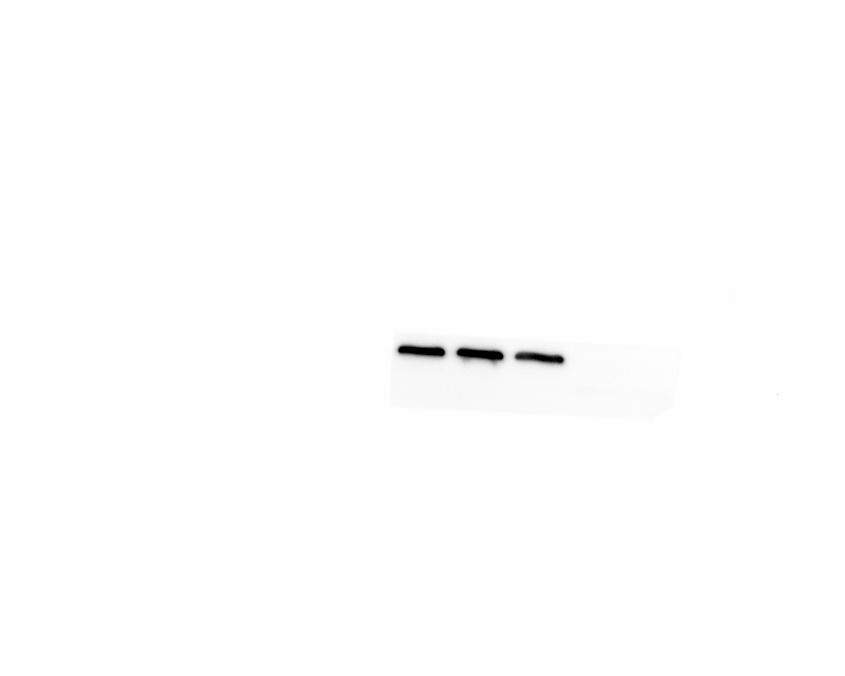

Supplement: Supplementary file 1 [file cancers-14-04645-s001.zip › cancers-1921606-original/westernblot_LLC_apoptosis/2021_12_23_actin/CHEMI_12232021_092413_(Chemi).jpg]

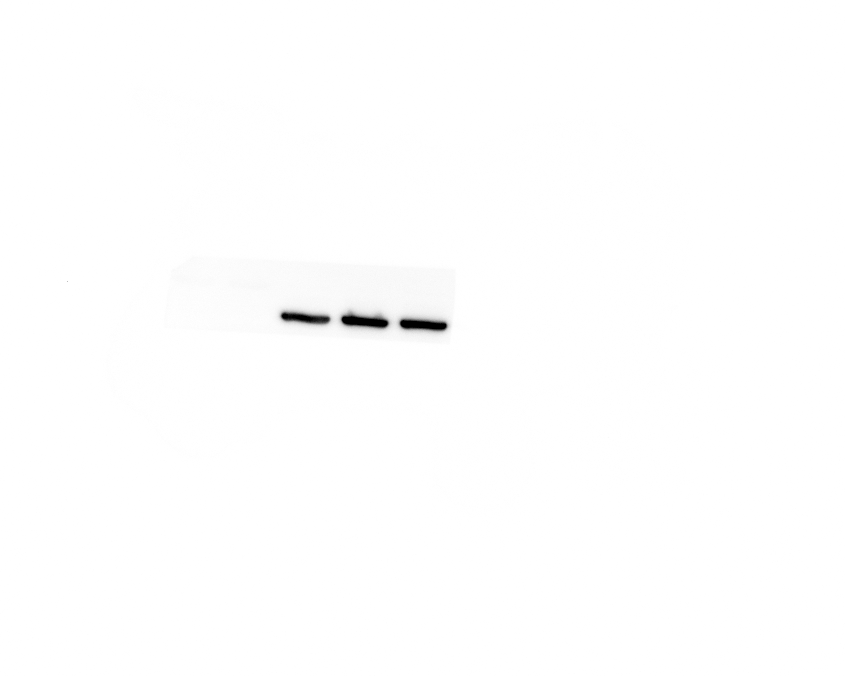

Supplement: Supplementary file 1 [file cancers-14-04645-s001.zip › cancers-1921606-original/westernblot_LLC_apoptosis/2021_12_23_actin/CHEMI_12232021_092413_(Chemi).tif]

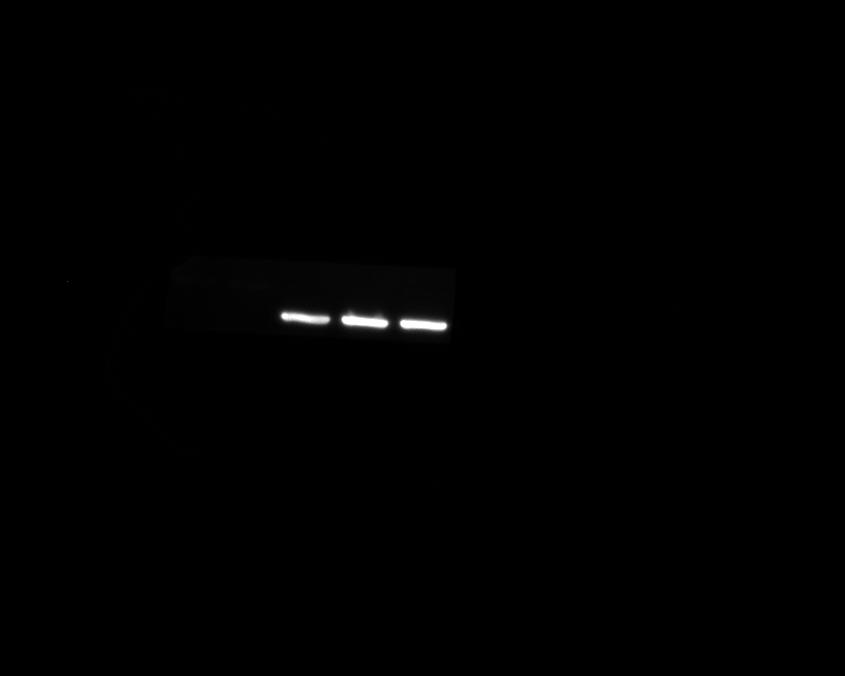

Supplement: Supplementary file 1 [file cancers-14-04645-s001.zip › cancers-1921606-original/westernblot_LLC_apoptosis/2021_12_23_actin/CHEMI_12232021_092413_(Chemi)_raw.tif]

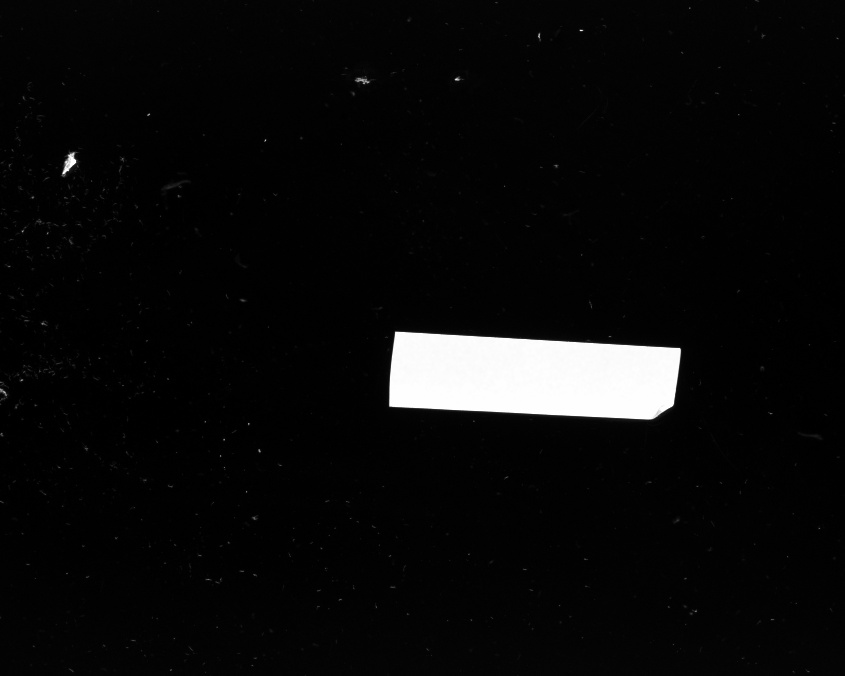

Supplement: Supplementary file 1 [file cancers-14-04645-s001.zip › cancers-1921606-original/westernblot_LLC_apoptosis/2021_12_23_actin/CHEMI_12232021_092413_(Membrane).jpg]

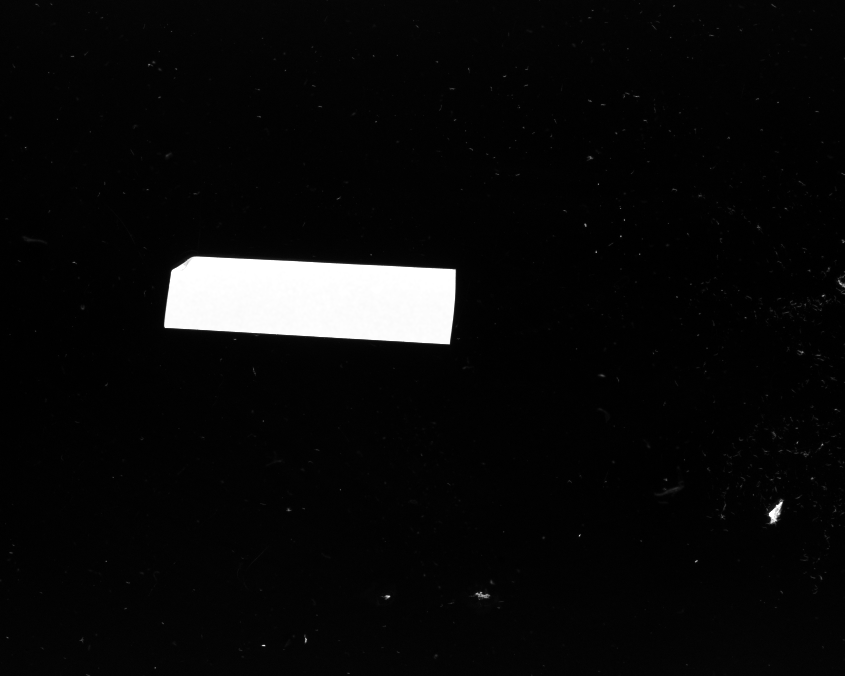

Supplement: Supplementary file 1 [file cancers-14-04645-s001.zip › cancers-1921606-original/westernblot_LLC_apoptosis/2021_12_23_actin/CHEMI_12232021_092413_(Membrane).tif]

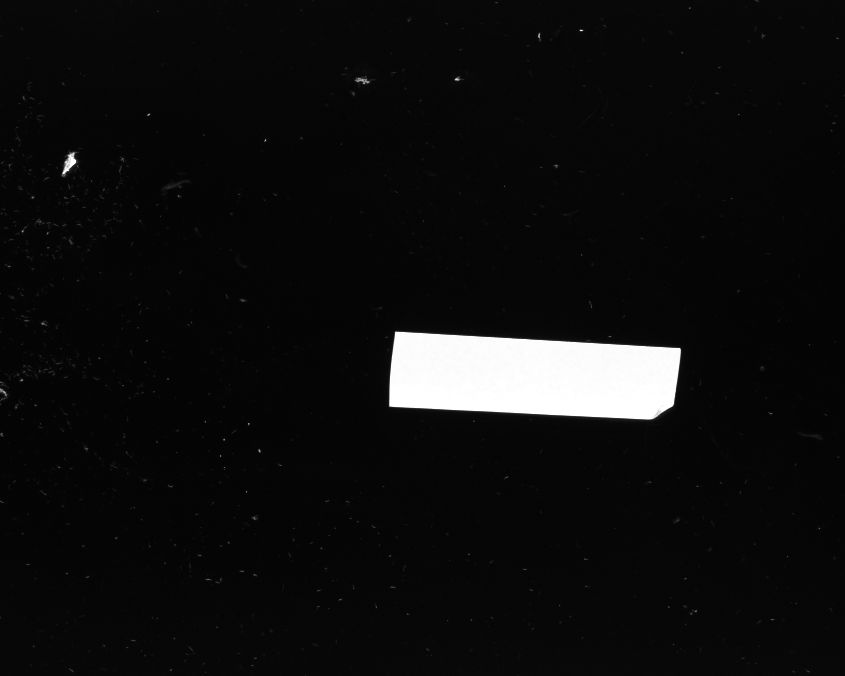

Supplement: Supplementary file 1 [file cancers-14-04645-s001.zip › cancers-1921606-original/westernblot_LLC_apoptosis/2021_12_23_actin/CHEMI_12232021_092413_(Membrane)_raw.tif]

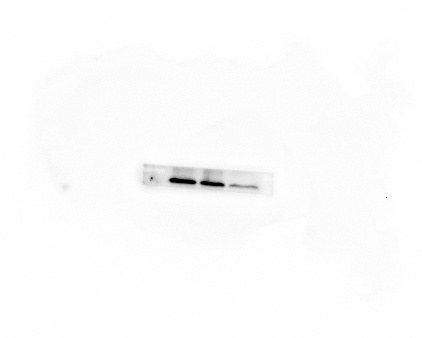

Supplement: Supplementary file 1 [file cancers-14-04645-s001.zip › cancers-1921606-original/westernblot_LLC_apoptosis/2021_12_23_bax/CHEMI_12232021_103624_(Chemi).jpg]

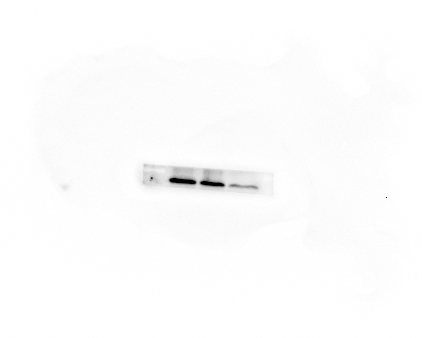

Supplement: Supplementary file 1 [file cancers-14-04645-s001.zip › cancers-1921606-original/westernblot_LLC_apoptosis/2021_12_23_bax/CHEMI_12232021_103624_(Chemi).tif]

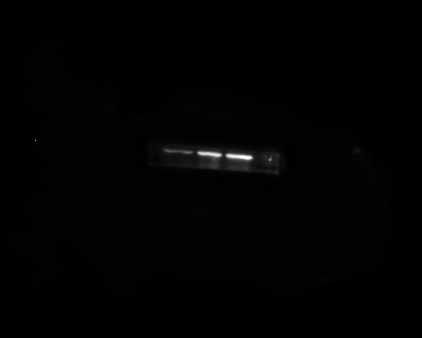

Supplement: Supplementary file 1 [file cancers-14-04645-s001.zip › cancers-1921606-original/westernblot_LLC_apoptosis/2021_12_23_bax/CHEMI_12232021_103624_(Chemi)_raw.tif]

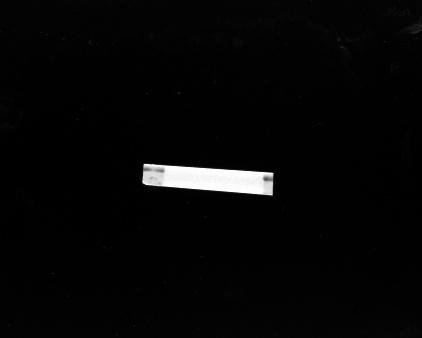

Supplement: Supplementary file 1 [file cancers-14-04645-s001.zip › cancers-1921606-original/westernblot_LLC_apoptosis/2021_12_23_bax/CHEMI_12232021_103624_(Membrane).jpg]

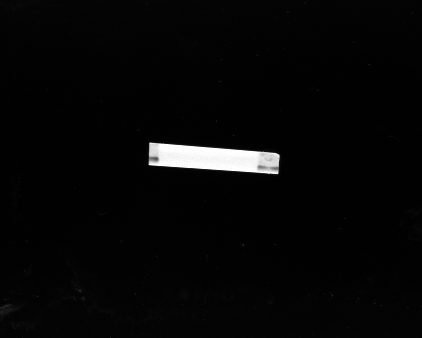

Supplement: Supplementary file 1 [file cancers-14-04645-s001.zip › cancers-1921606-original/westernblot_LLC_apoptosis/2021_12_23_bax/CHEMI_12232021_103624_(Membrane).tif]

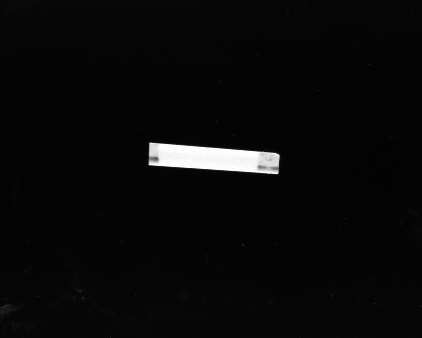

Supplement: Supplementary file 1 [file cancers-14-04645-s001.zip › cancers-1921606-original/westernblot_LLC_apoptosis/2021_12_23_bax/CHEMI_12232021_103624_(Membrane)_raw.tif]

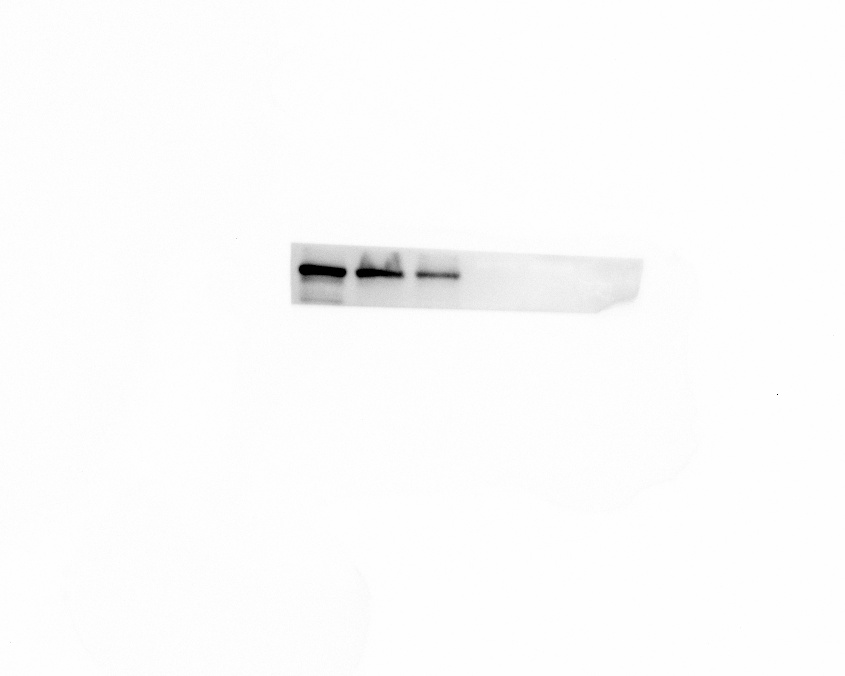

Supplement: Supplementary file 1 [file cancers-14-04645-s001.zip › cancers-1921606-original/westernblot_LLC_apoptosis/2021_12_23_bcl2/CHEMI_12232021_095151_(Chemi).jpg]

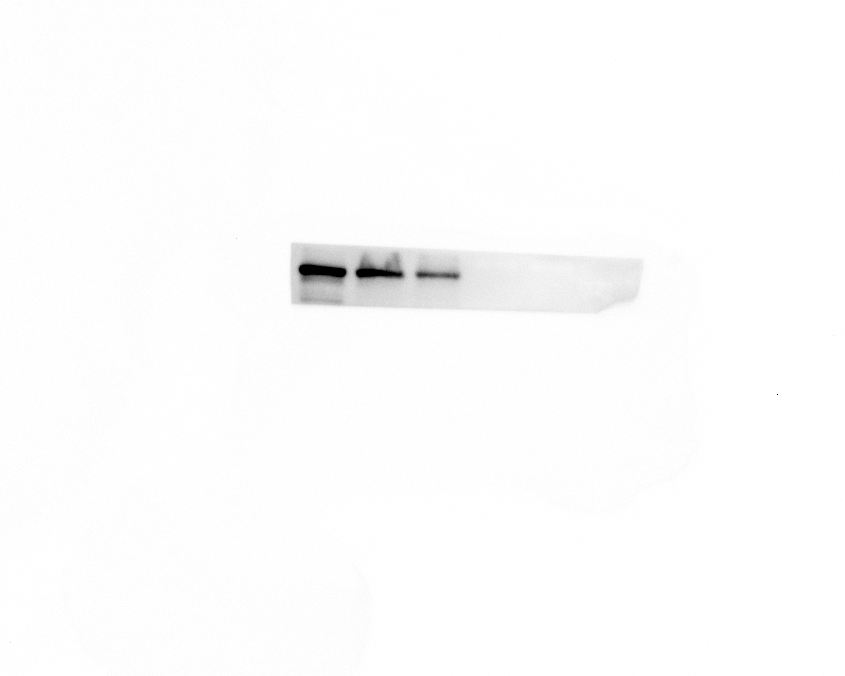

Supplement: Supplementary file 1 [file cancers-14-04645-s001.zip › cancers-1921606-original/westernblot_LLC_apoptosis/2021_12_23_bcl2/CHEMI_12232021_095151_(Chemi).tif]

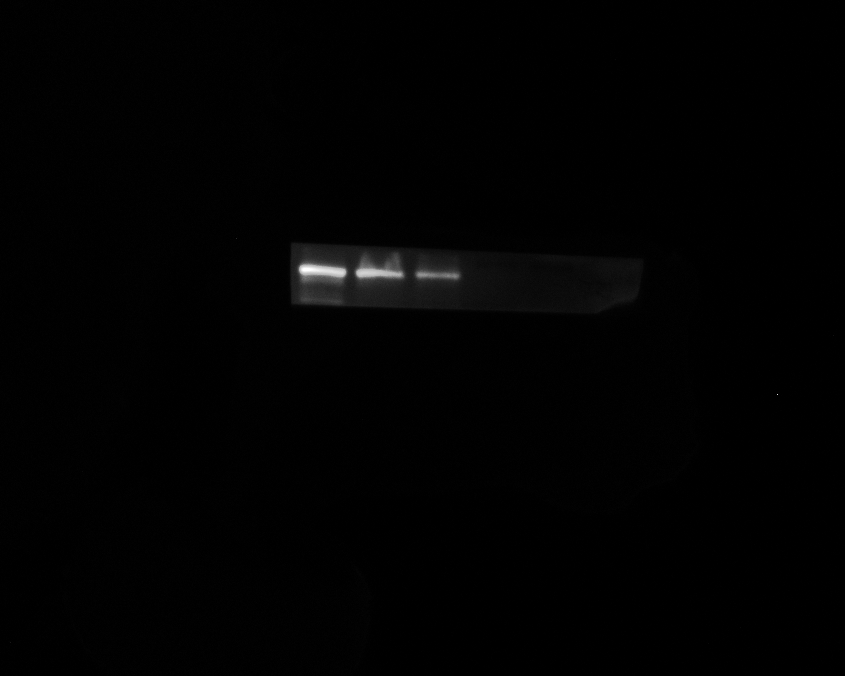

Supplement: Supplementary file 1 [file cancers-14-04645-s001.zip › cancers-1921606-original/westernblot_LLC_apoptosis/2021_12_23_bcl2/CHEMI_12232021_095151_(Chemi)_raw.tif]

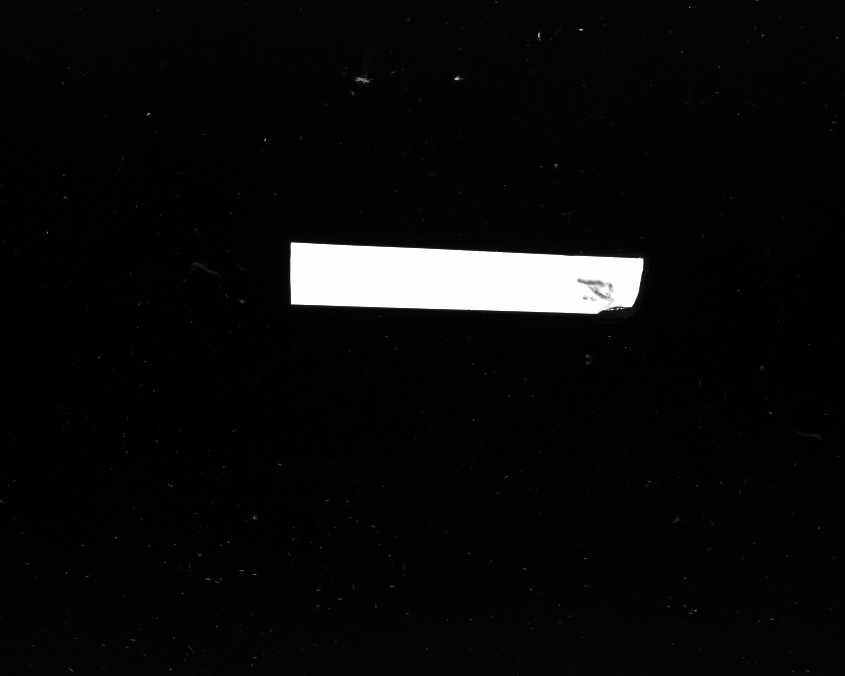

Supplement: Supplementary file 1 [file cancers-14-04645-s001.zip › cancers-1921606-original/westernblot_LLC_apoptosis/2021_12_23_bcl2/CHEMI_12232021_095151_(Membrane).jpg]

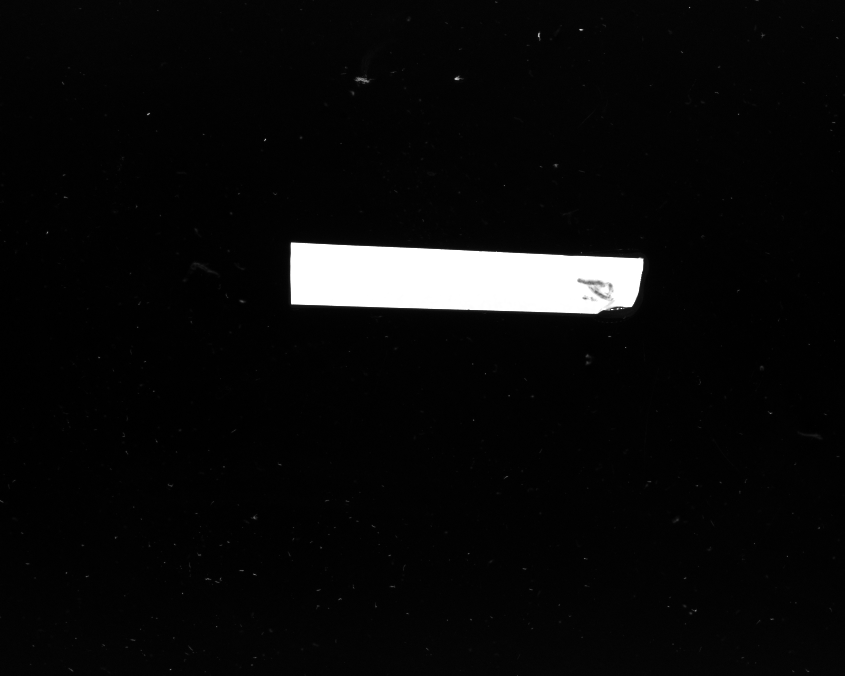

Supplement: Supplementary file 1 [file cancers-14-04645-s001.zip › cancers-1921606-original/westernblot_LLC_apoptosis/2021_12_23_bcl2/CHEMI_12232021_095151_(Membrane).tif]

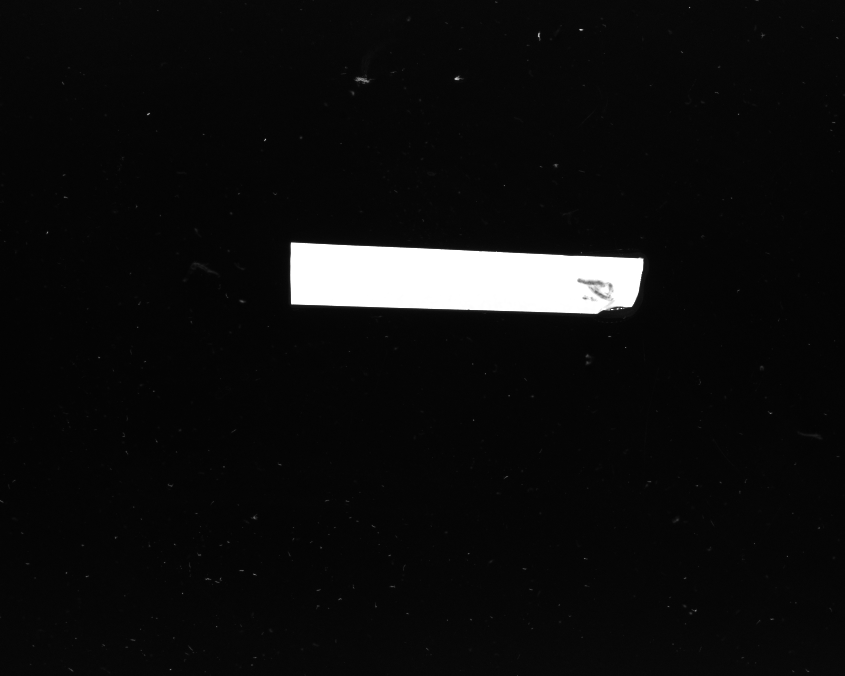

Supplement: Supplementary file 1 [file cancers-14-04645-s001.zip › cancers-1921606-original/westernblot_LLC_apoptosis/2021_12_23_bcl2/CHEMI_12232021_095151_(Membrane)_raw.tif]

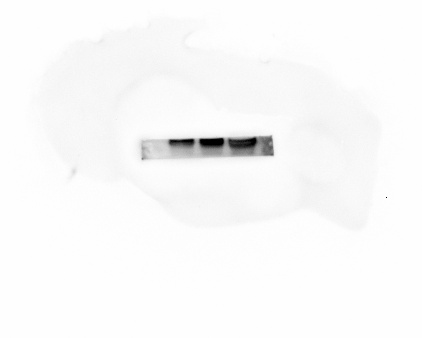

Supplement: Supplementary file 1 [file cancers-14-04645-s001.zip › cancers-1921606-original/westernblot_LLC_apoptosis/2021_12_23_ca9/CHEMI_01142021_181512_(Chemi).jpg]

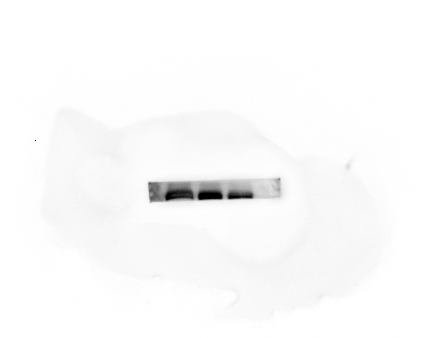

Supplement: Supplementary file 1 [file cancers-14-04645-s001.zip › cancers-1921606-original/westernblot_LLC_apoptosis/2021_12_23_ca9/CHEMI_01142021_181512_(Chemi).tif]

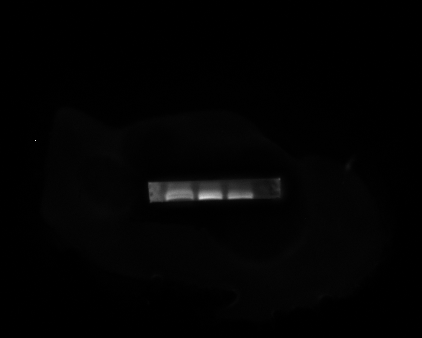

Supplement: Supplementary file 1 [file cancers-14-04645-s001.zip › cancers-1921606-original/westernblot_LLC_apoptosis/2021_12_23_ca9/CHEMI_01142021_181512_(Chemi)_raw.tif]

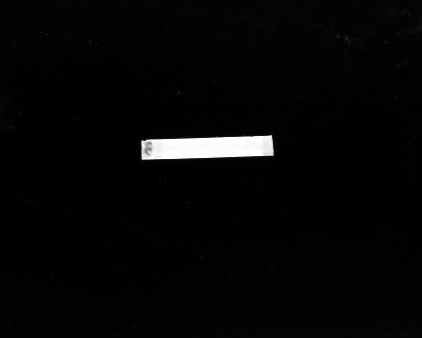

Supplement: Supplementary file 1 [file cancers-14-04645-s001.zip › cancers-1921606-original/westernblot_LLC_apoptosis/2021_12_23_ca9/CHEMI_01142021_181512_(Membrane).jpg]

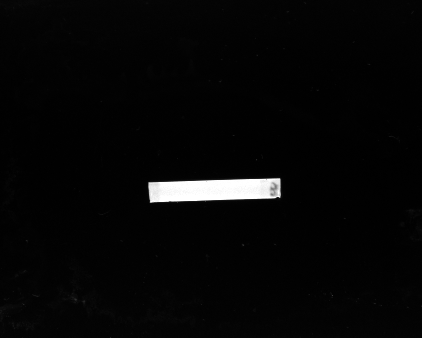

Supplement: Supplementary file 1 [file cancers-14-04645-s001.zip › cancers-1921606-original/westernblot_LLC_apoptosis/2021_12_23_ca9/CHEMI_01142021_181512_(Membrane).tif]

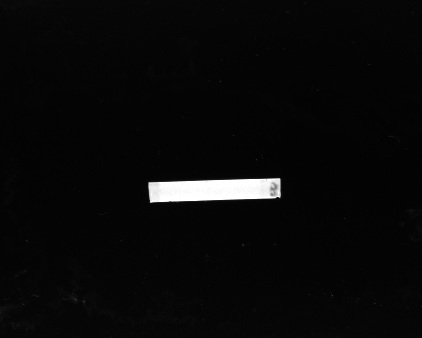

Supplement: Supplementary file 1 [file cancers-14-04645-s001.zip › cancers-1921606-original/westernblot_LLC_apoptosis/2021_12_23_ca9/CHEMI_01142021_181512_(Membrane)_raw.tif]

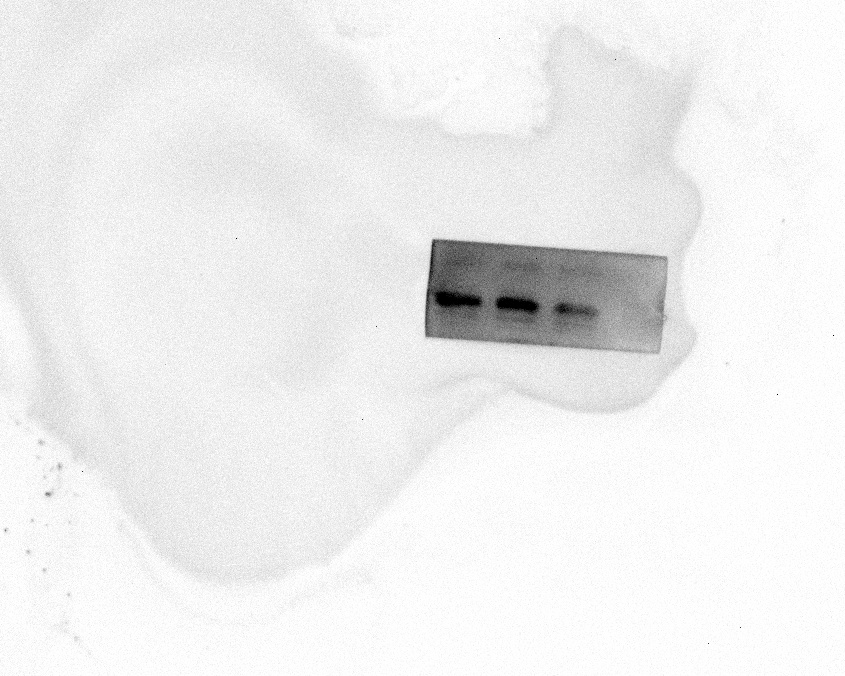

Supplement: Supplementary file 1 [file cancers-14-04645-s001.zip › cancers-1921606-original/westernblot_LLC_apoptosis/2021_12_23_parp1/CHEMI_12232021_103248_(Chemi).jpg]

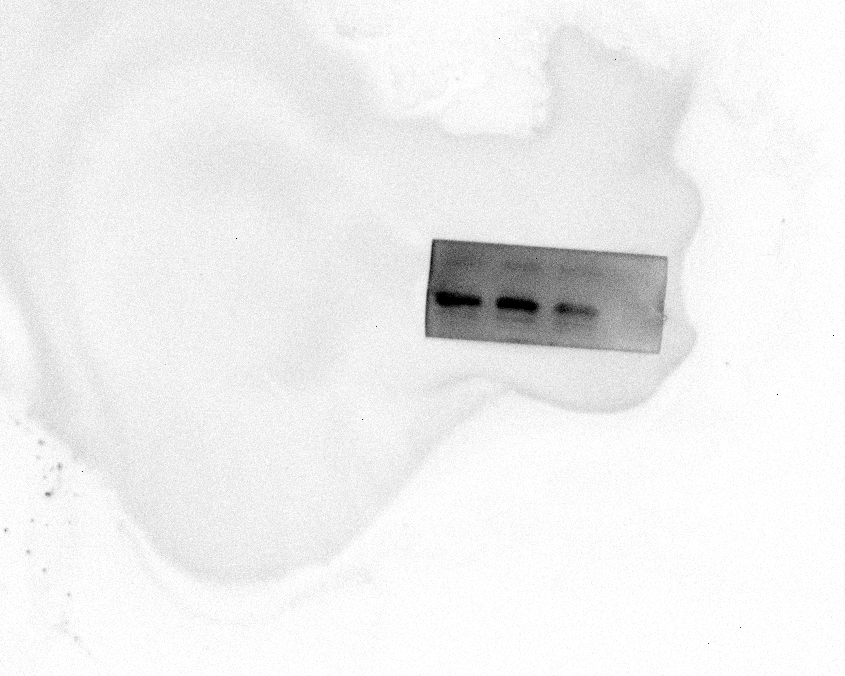

Supplement: Supplementary file 1 [file cancers-14-04645-s001.zip › cancers-1921606-original/westernblot_LLC_apoptosis/2021_12_23_parp1/CHEMI_12232021_103248_(Chemi).tif]

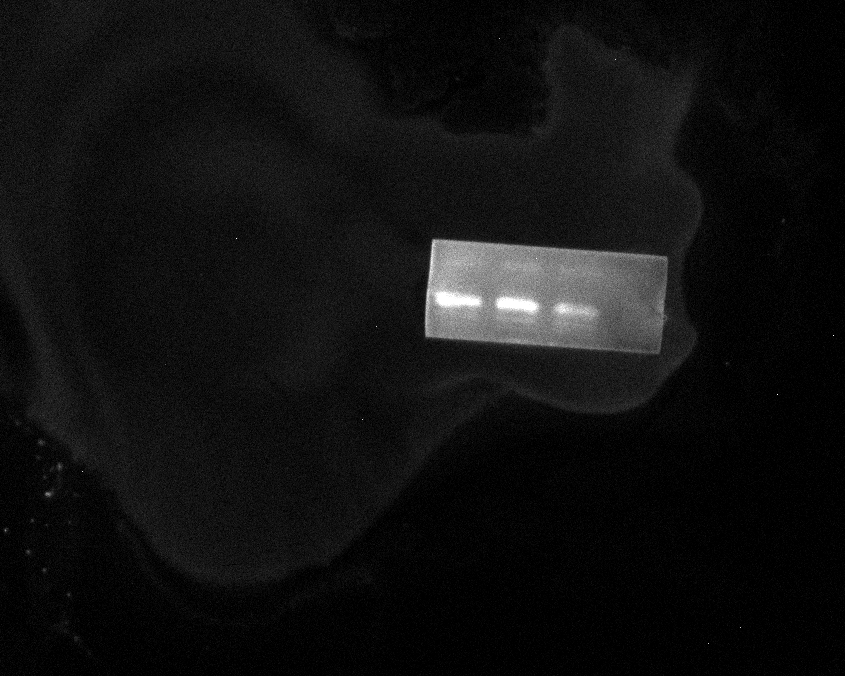

Supplement: Supplementary file 1 [file cancers-14-04645-s001.zip › cancers-1921606-original/westernblot_LLC_apoptosis/2021_12_23_parp1/CHEMI_12232021_103248_(Chemi)_raw.tif]

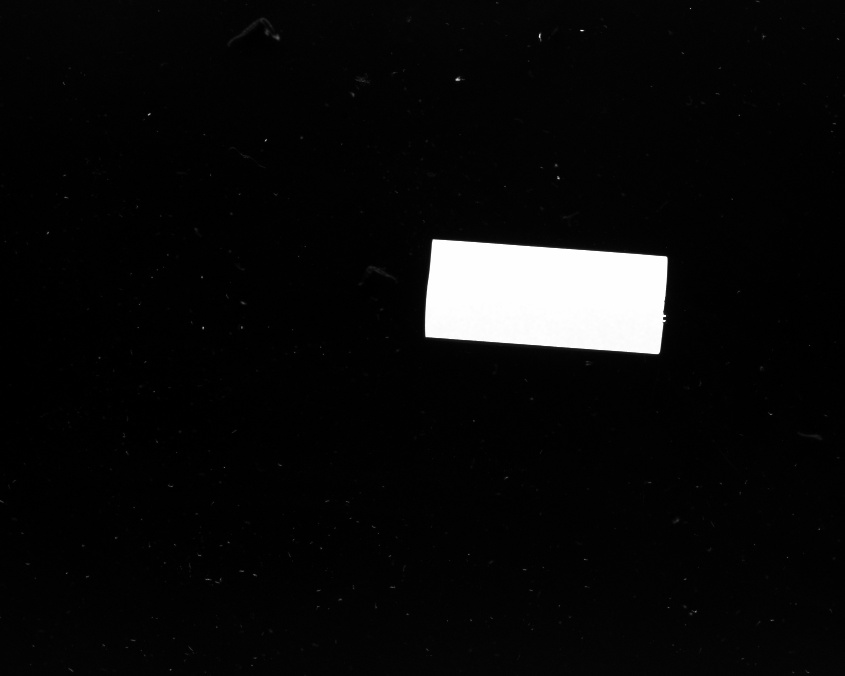

Supplement: Supplementary file 1 [file cancers-14-04645-s001.zip › cancers-1921606-original/westernblot_LLC_apoptosis/2021_12_23_parp1/CHEMI_12232021_103248_(Membrane).jpg]

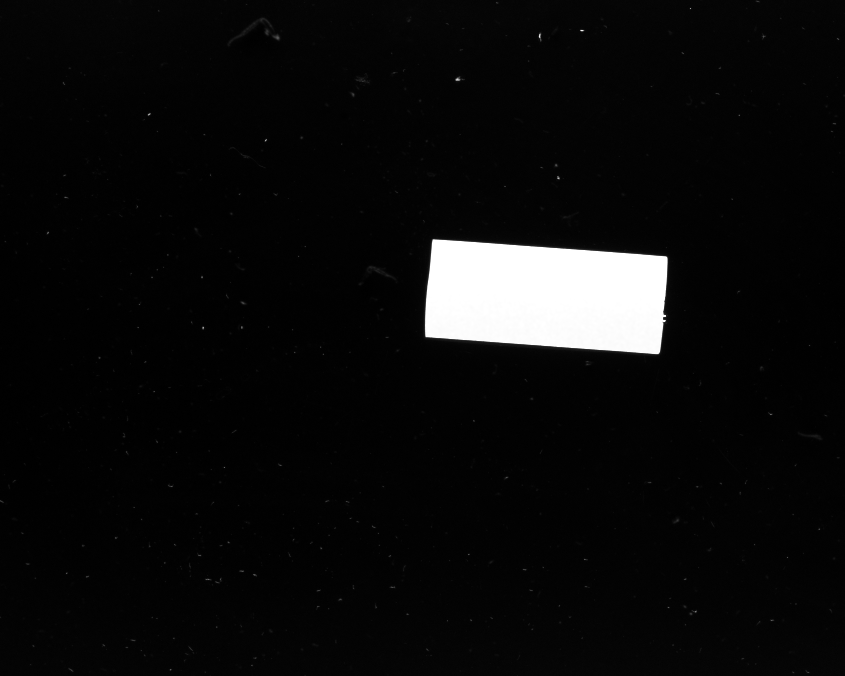

Supplement: Supplementary file 1 [file cancers-14-04645-s001.zip › cancers-1921606-original/westernblot_LLC_apoptosis/2021_12_23_parp1/CHEMI_12232021_103248_(Membrane).tif]

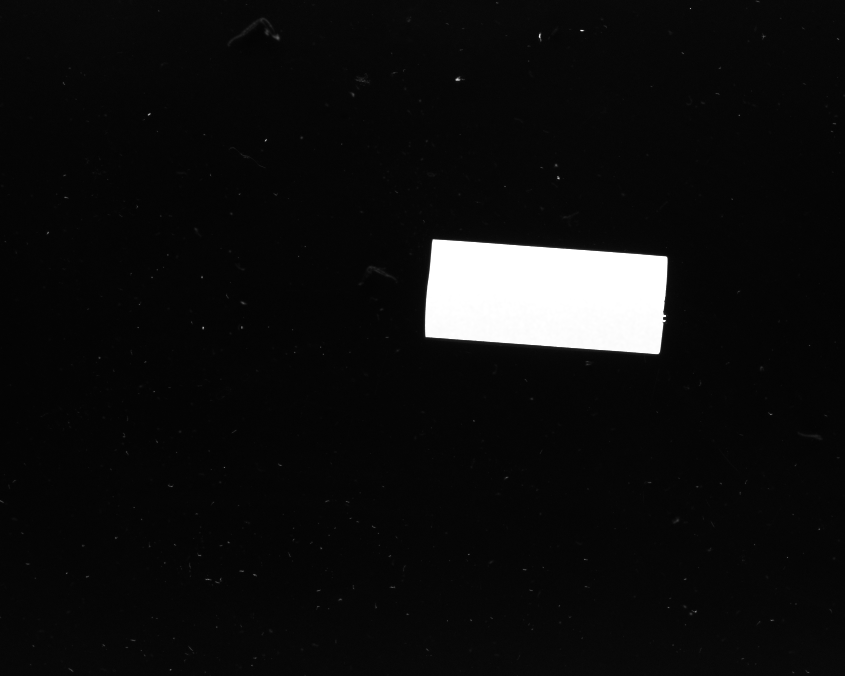

Supplement: Supplementary file 1 [file cancers-14-04645-s001.zip › cancers-1921606-original/westernblot_LLC_apoptosis/2021_12_23_parp1/CHEMI_12232021_103248_(Membrane)_raw.tif]

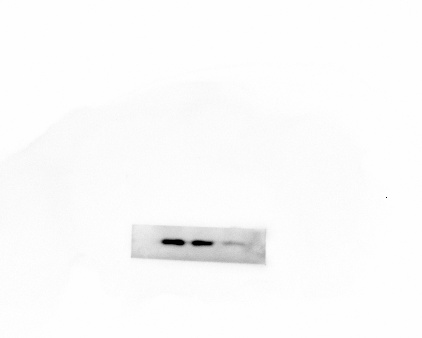

Supplement: Supplementary file 1 [file cancers-14-04645-s001.zip › cancers-1921606-original/westernblot_LLC_apoptosis/2021_12_24_cytc/CHEMI_01142021_174310_(Chemi).jpg]

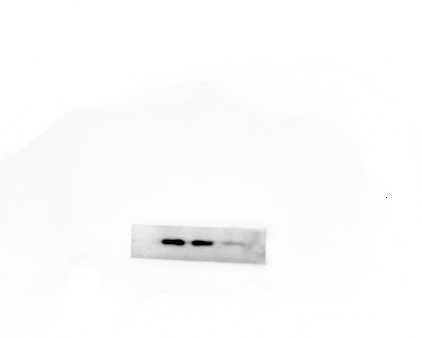

Supplement: Supplementary file 1 [file cancers-14-04645-s001.zip › cancers-1921606-original/westernblot_LLC_apoptosis/2021_12_24_cytc/CHEMI_01142021_174310_(Chemi).tif]

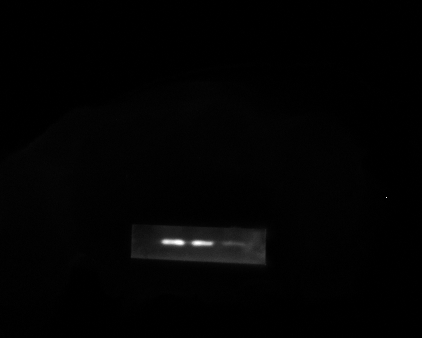

Supplement: Supplementary file 1 [file cancers-14-04645-s001.zip › cancers-1921606-original/westernblot_LLC_apoptosis/2021_12_24_cytc/CHEMI_01142021_174310_(Chemi)_raw.tif]

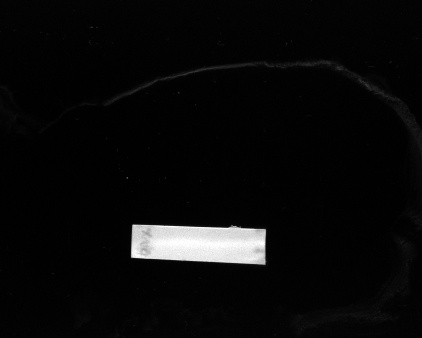

Supplement: Supplementary file 1 [file cancers-14-04645-s001.zip › cancers-1921606-original/westernblot_LLC_apoptosis/2021_12_24_cytc/CHEMI_01142021_174310_(Membrane).jpg]

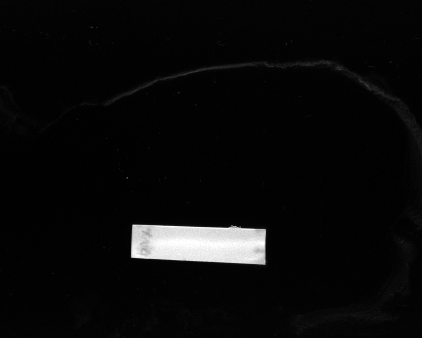

Supplement: Supplementary file 1 [file cancers-14-04645-s001.zip › cancers-1921606-original/westernblot_LLC_apoptosis/2021_12_24_cytc/CHEMI_01142021_174310_(Membrane).tif]

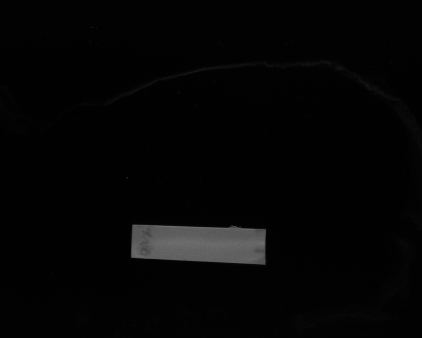

Supplement: Supplementary file 1 [file cancers-14-04645-s001.zip › cancers-1921606-original/westernblot_LLC_apoptosis/2021_12_24_cytc/CHEMI_01142021_174310_(Membrane)_raw.tif]

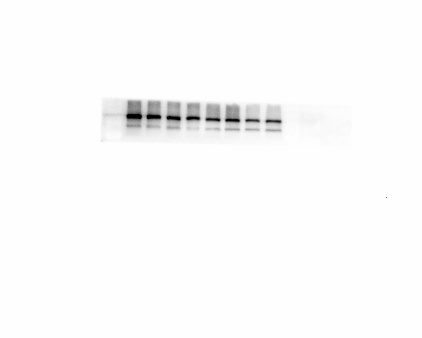

Supplement: Supplementary file 1 [file cancers-14-04645-s001.zip › cancers-1921606-original/westernblot_SPCA1_apoptosis/2021_07_13_parp1/CHEMI_07132021_163536_(Chemi).jpg]

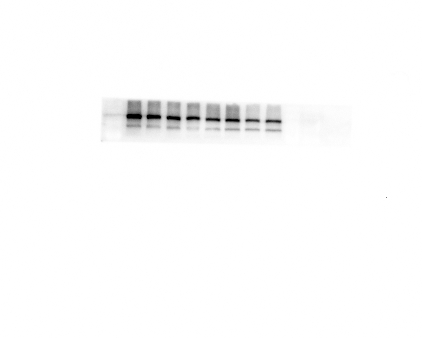

Supplement: Supplementary file 1 [file cancers-14-04645-s001.zip › cancers-1921606-original/westernblot_SPCA1_apoptosis/2021_07_13_parp1/CHEMI_07132021_163536_(Chemi).tif]

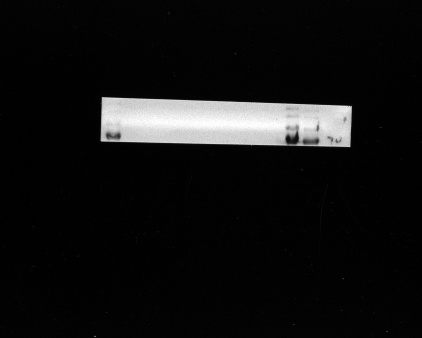

Supplement: Supplementary file 1 [file cancers-14-04645-s001.zip › cancers-1921606-original/westernblot_SPCA1_apoptosis/2021_07_13_parp1/CHEMI_07132021_163536_(Membrane).jpg]

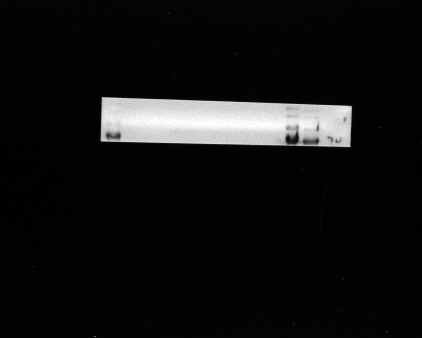

Supplement: Supplementary file 1 [file cancers-14-04645-s001.zip › cancers-1921606-original/westernblot_SPCA1_apoptosis/2021_07_13_parp1/CHEMI_07132021_163536_(Membrane).tif]

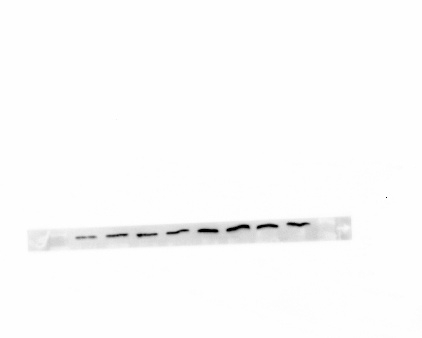

Supplement: Supplementary file 1 [file cancers-14-04645-s001.zip › cancers-1921606-original/westernblot_SPCA1_apoptosis/2021_08_15_bcl2/CHEMI_08152021_150231_(Chemi).jpg]

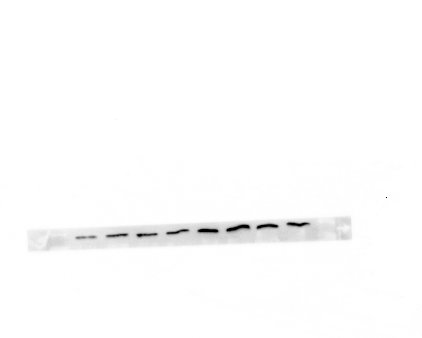

Supplement: Supplementary file 1 [file cancers-14-04645-s001.zip › cancers-1921606-original/westernblot_SPCA1_apoptosis/2021_08_15_bcl2/CHEMI_08152021_150231_(Chemi).png]

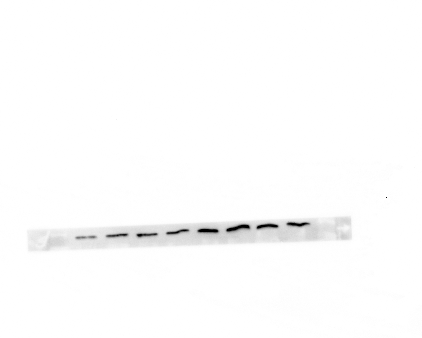

Supplement: Supplementary file 1 [file cancers-14-04645-s001.zip › cancers-1921606-original/westernblot_SPCA1_apoptosis/2021_08_15_bcl2/CHEMI_08152021_150231_(Chemi).tif]

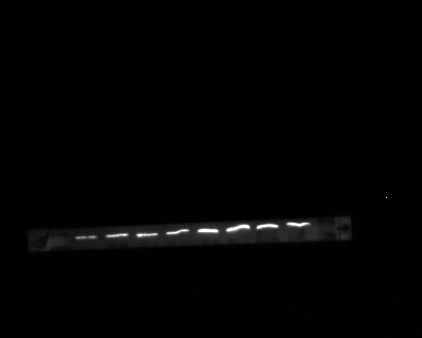

Supplement: Supplementary file 1 [file cancers-14-04645-s001.zip › cancers-1921606-original/westernblot_SPCA1_apoptosis/2021_08_15_bcl2/CHEMI_08152021_150231_(Chemi)_raw.tif]

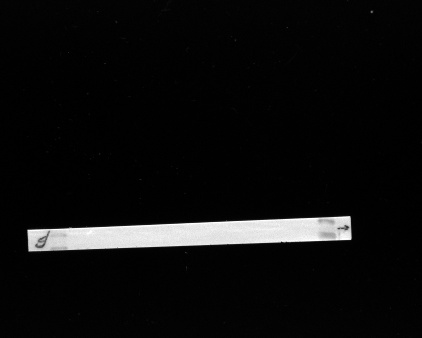

Supplement: Supplementary file 1 [file cancers-14-04645-s001.zip › cancers-1921606-original/westernblot_SPCA1_apoptosis/2021_08_15_bcl2/CHEMI_08152021_150231_(Membrane).jpg]

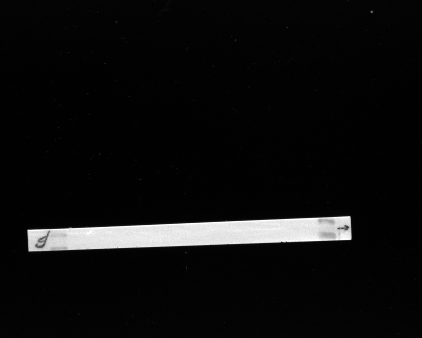

Supplement: Supplementary file 1 [file cancers-14-04645-s001.zip › cancers-1921606-original/westernblot_SPCA1_apoptosis/2021_08_15_bcl2/CHEMI_08152021_150231_(Membrane).png]

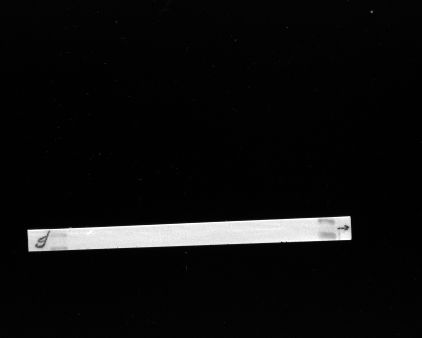

Supplement: Supplementary file 1 [file cancers-14-04645-s001.zip › cancers-1921606-original/westernblot_SPCA1_apoptosis/2021_08_15_bcl2/CHEMI_08152021_150231_(Membrane).tif]

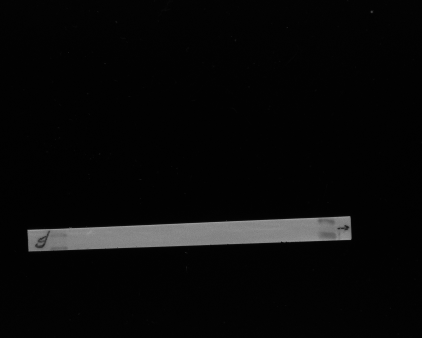

Supplement: Supplementary file 1 [file cancers-14-04645-s001.zip › cancers-1921606-original/westernblot_SPCA1_apoptosis/2021_08_15_bcl2/CHEMI_08152021_150231_(Membrane)_raw.tif]

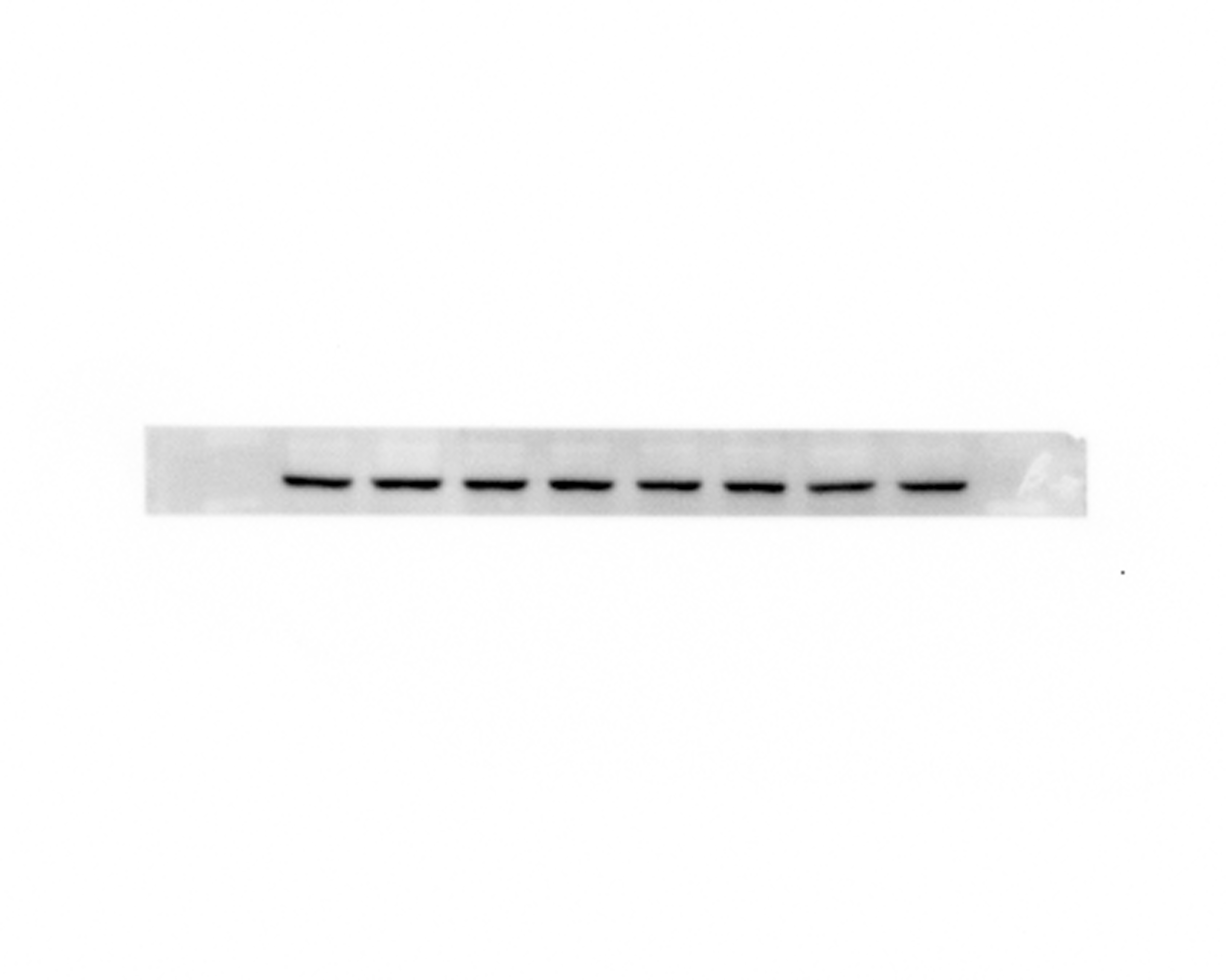

Supplement: Supplementary file 1 [file cancers-14-04645-s001.zip › cancers-1921606-original/westernblot_SPCA1_apoptosis/2021_08_28_actin/CHEMI_08282021_203035_(Chemi).tif]

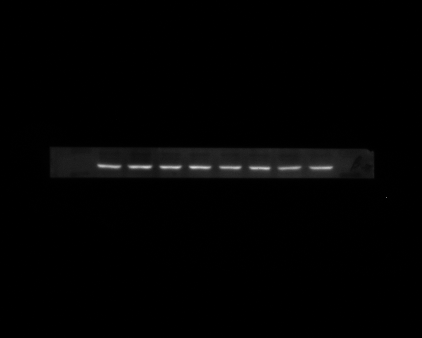

Supplement: Supplementary file 1 [file cancers-14-04645-s001.zip › cancers-1921606-original/westernblot_SPCA1_apoptosis/2021_08_28_actin/CHEMI_08282021_203035_(Chemi)_raw.tif]

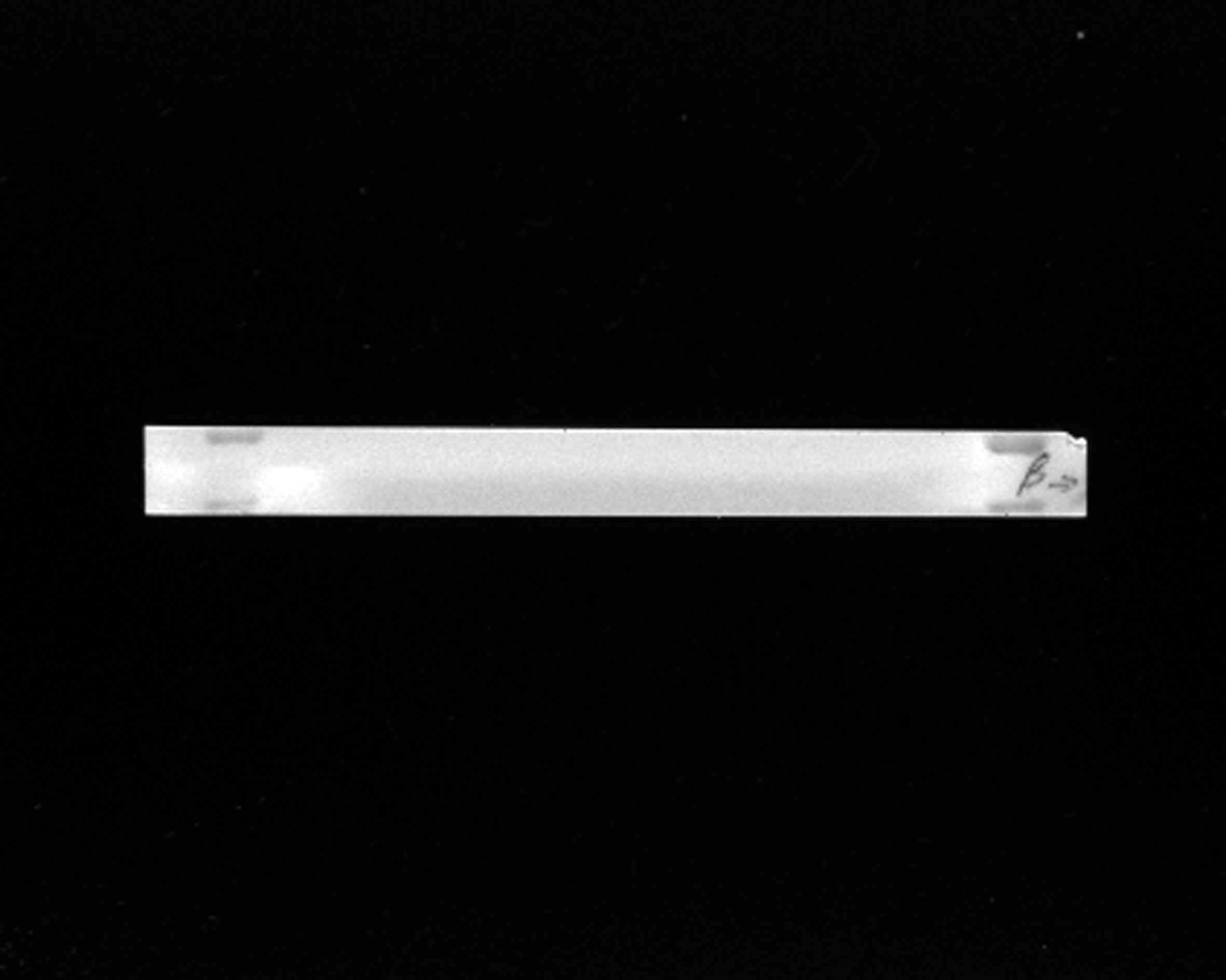

Supplement: Supplementary file 1 [file cancers-14-04645-s001.zip › cancers-1921606-original/westernblot_SPCA1_apoptosis/2021_08_28_actin/CHEMI_08282021_203035_(Membrane).tif]

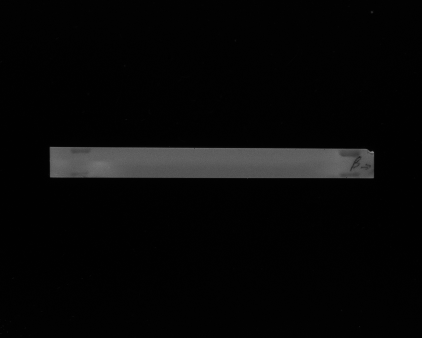

Supplement: Supplementary file 1 [file cancers-14-04645-s001.zip › cancers-1921606-original/westernblot_SPCA1_apoptosis/2021_08_28_actin/CHEMI_08282021_203035_(Membrane)_raw.tif]

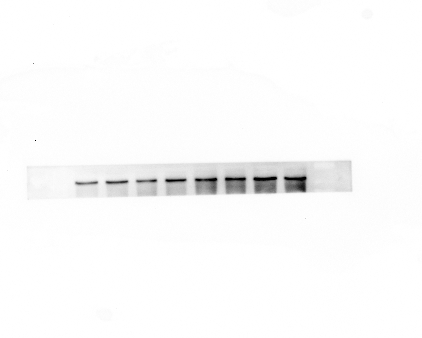

Supplement: Supplementary file 1 [file cancers-14-04645-s001.zip › cancers-1921606-original/westernblot_SPCA1_apoptosis/2021_08_28_bax/CHEMI_08282021_203622_(Chemi).tif]

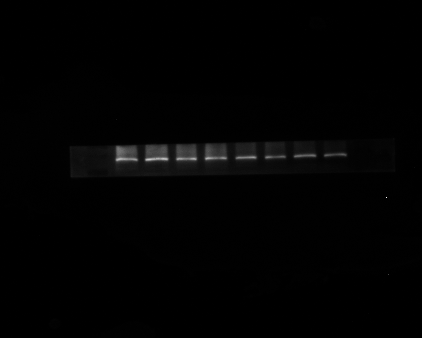

Supplement: Supplementary file 1 [file cancers-14-04645-s001.zip › cancers-1921606-original/westernblot_SPCA1_apoptosis/2021_08_28_bax/CHEMI_08282021_203622_(Chemi)_raw.tif]

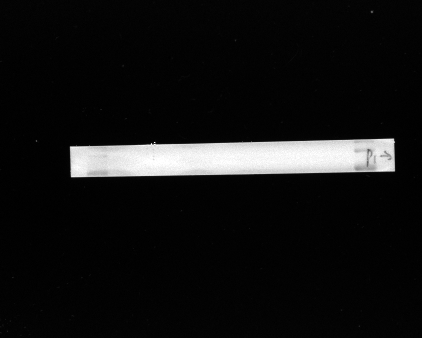

Supplement: Supplementary file 1 [file cancers-14-04645-s001.zip › cancers-1921606-original/westernblot_SPCA1_apoptosis/2021_08_28_bax/CHEMI_08282021_203622_(Membrane).tif]

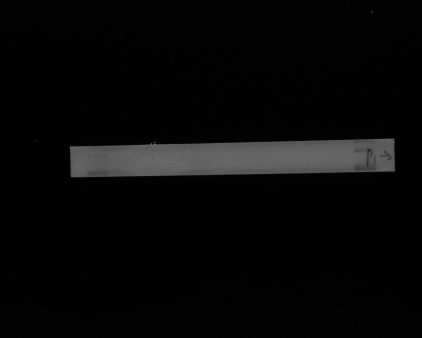

Supplement: Supplementary file 1 [file cancers-14-04645-s001.zip › cancers-1921606-original/westernblot_SPCA1_apoptosis/2021_08_28_bax/CHEMI_08282021_203622_(Membrane)_raw.tif]

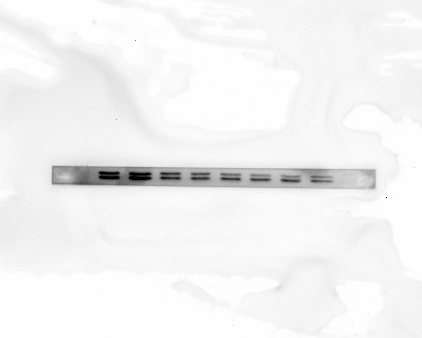

Supplement: Supplementary file 1 [file cancers-14-04645-s001.zip › cancers-1921606-original/westernblot_SPCA1_apoptosis/2021_08_28_c9/CHEMI_08152021_151135_(Chemi).jpg]

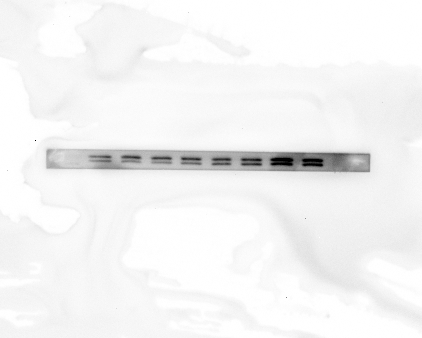

Supplement: Supplementary file 1 [file cancers-14-04645-s001.zip › cancers-1921606-original/westernblot_SPCA1_apoptosis/2021_08_28_c9/CHEMI_08152021_151135_(Chemi).png]

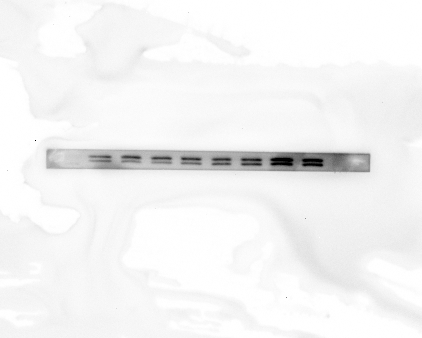

Supplement: Supplementary file 1 [file cancers-14-04645-s001.zip › cancers-1921606-original/westernblot_SPCA1_apoptosis/2021_08_28_c9/CHEMI_08152021_151135_(Chemi).tif]

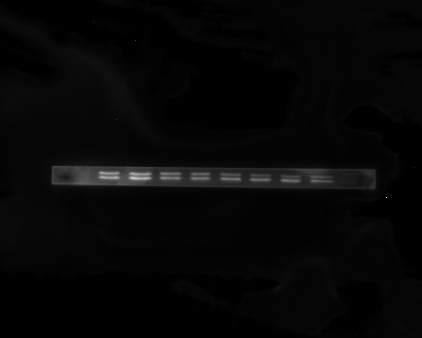

Supplement: Supplementary file 1 [file cancers-14-04645-s001.zip › cancers-1921606-original/westernblot_SPCA1_apoptosis/2021_08_28_c9/CHEMI_08152021_151135_(Chemi)_raw.tif]

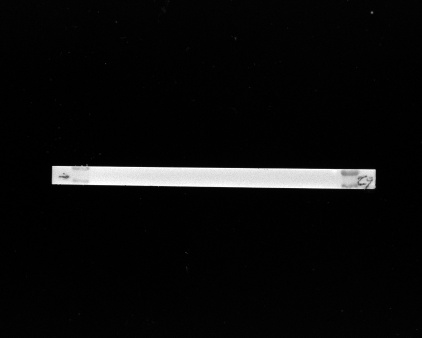

Supplement: Supplementary file 1 [file cancers-14-04645-s001.zip › cancers-1921606-original/westernblot_SPCA1_apoptosis/2021_08_28_c9/CHEMI_08152021_151135_(Membrane).jpg]

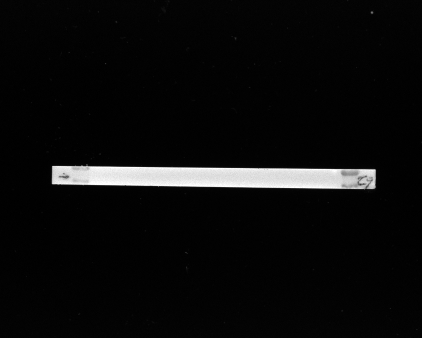

Supplement: Supplementary file 1 [file cancers-14-04645-s001.zip › cancers-1921606-original/westernblot_SPCA1_apoptosis/2021_08_28_c9/CHEMI_08152021_151135_(Membrane).png]

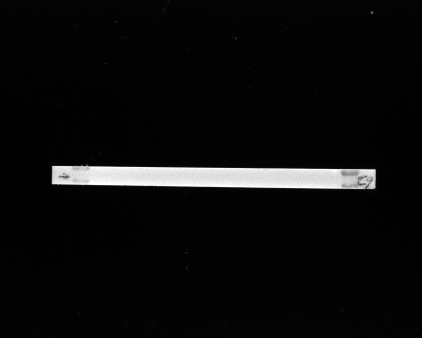

Supplement: Supplementary file 1 [file cancers-14-04645-s001.zip › cancers-1921606-original/westernblot_SPCA1_apoptosis/2021_08_28_c9/CHEMI_08152021_151135_(Membrane).tif]

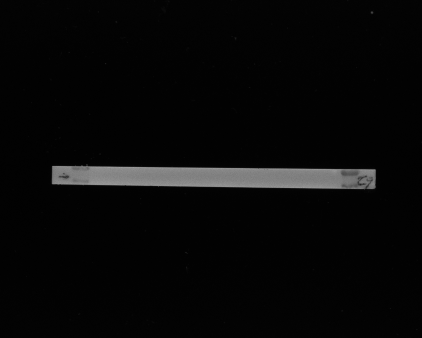

Supplement: Supplementary file 1 [file cancers-14-04645-s001.zip › cancers-1921606-original/westernblot_SPCA1_apoptosis/2021_08_28_c9/CHEMI_08152021_151135_(Membrane)_raw.tif]

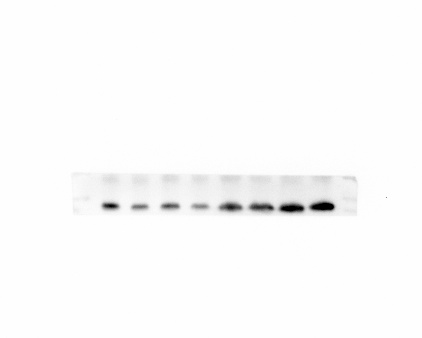

Supplement: Supplementary file 1 [file cancers-14-04645-s001.zip › cancers-1921606-original/westernblot_SPCA1_apoptosis/2022_06_14_cytc/CHEMI_06142022_175543_(Chemi).jpg]

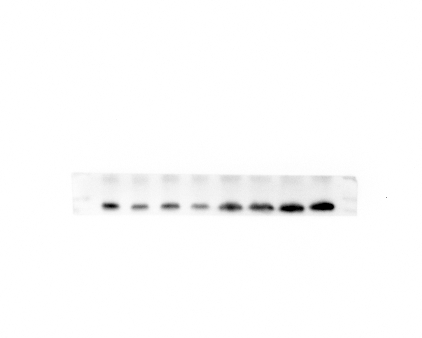

Supplement: Supplementary file 1 [file cancers-14-04645-s001.zip › cancers-1921606-original/westernblot_SPCA1_apoptosis/2022_06_14_cytc/CHEMI_06142022_175543_(Chemi).tif]

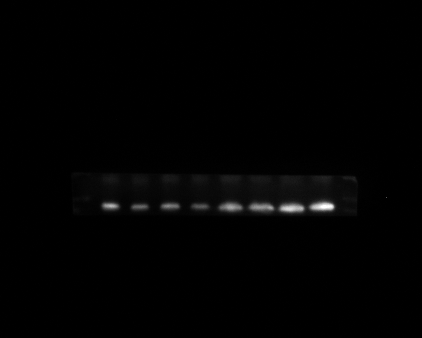

Supplement: Supplementary file 1 [file cancers-14-04645-s001.zip › cancers-1921606-original/westernblot_SPCA1_apoptosis/2022_06_14_cytc/CHEMI_06142022_175543_(Chemi)_raw.tif]

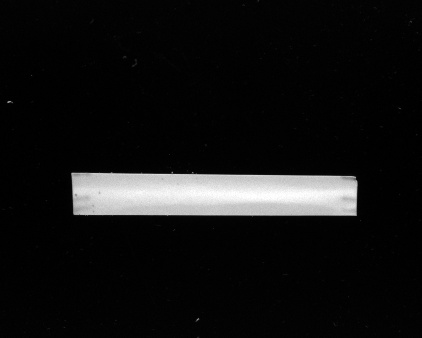

Supplement: Supplementary file 1 [file cancers-14-04645-s001.zip › cancers-1921606-original/westernblot_SPCA1_apoptosis/2022_06_14_cytc/CHEMI_06142022_175543_(Membrane).jpg]

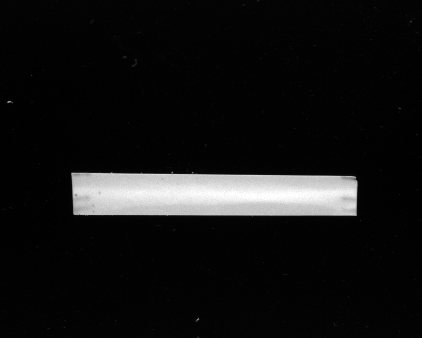

Supplement: Supplementary file 1 [file cancers-14-04645-s001.zip › cancers-1921606-original/westernblot_SPCA1_apoptosis/2022_06_14_cytc/CHEMI_06142022_175543_(Membrane).tif]

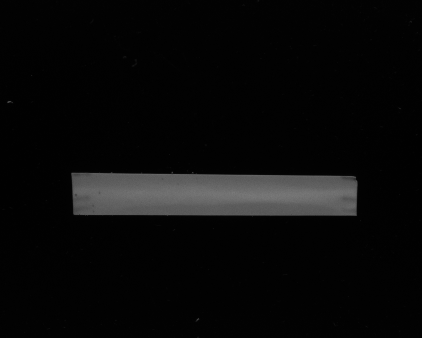

Supplement: Supplementary file 1 [file cancers-14-04645-s001.zip › cancers-1921606-original/westernblot_SPCA1_apoptosis/2022_06_14_cytc/CHEMI_06142022_175543_(Membrane)_raw.tif]

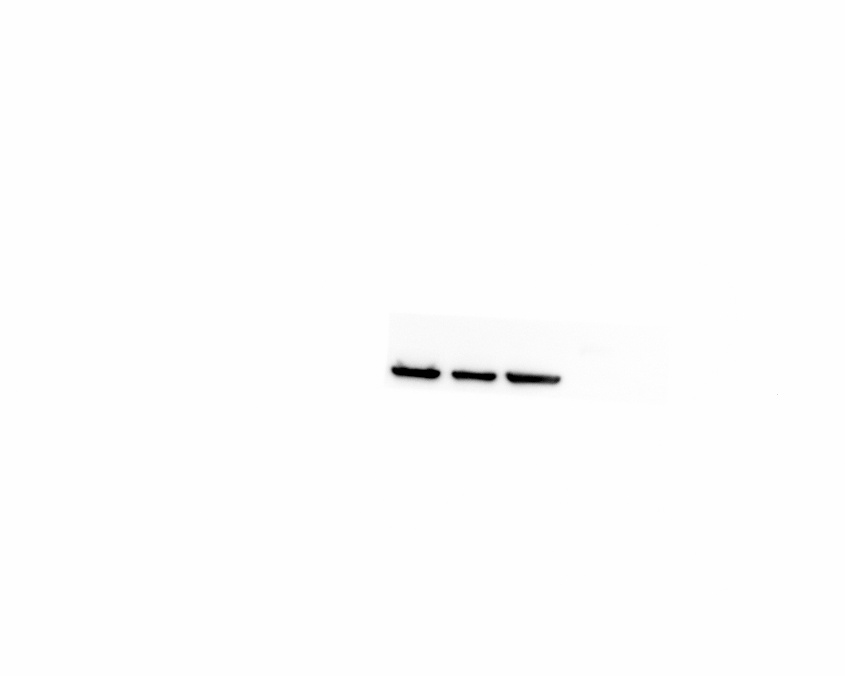

Supplement: Supplementary file 1 [file cancers-14-04645-s001.zip › cancers-1921606-original/westernblot_SPCA1_EMT/2021_01_14_actin/CHEMI_12232021_092323_(Chemi).jpg]

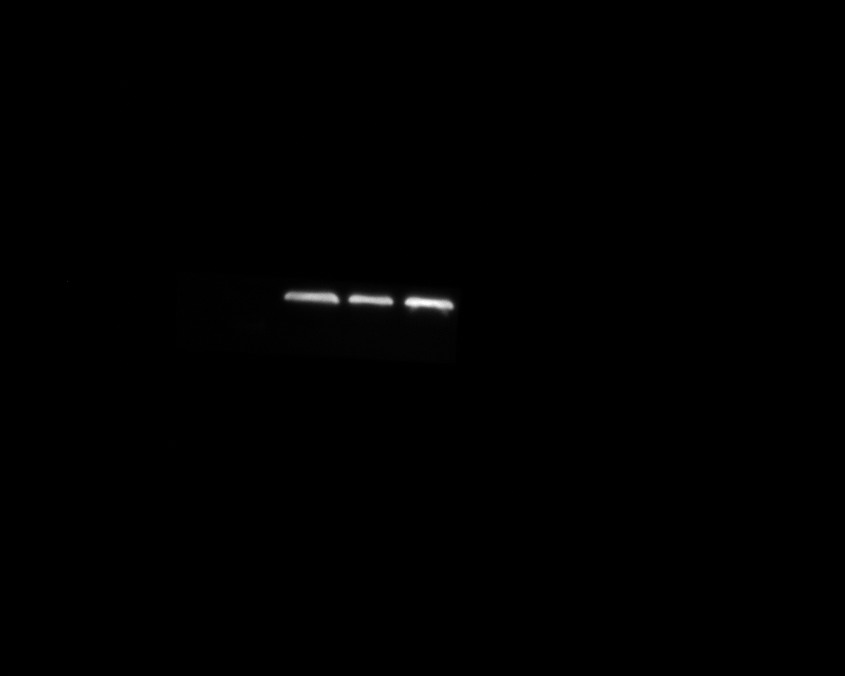

Supplement: Supplementary file 1 [file cancers-14-04645-s001.zip › cancers-1921606-original/westernblot_SPCA1_EMT/2021_01_14_actin/CHEMI_12232021_092323_(Chemi)_raw.tif]

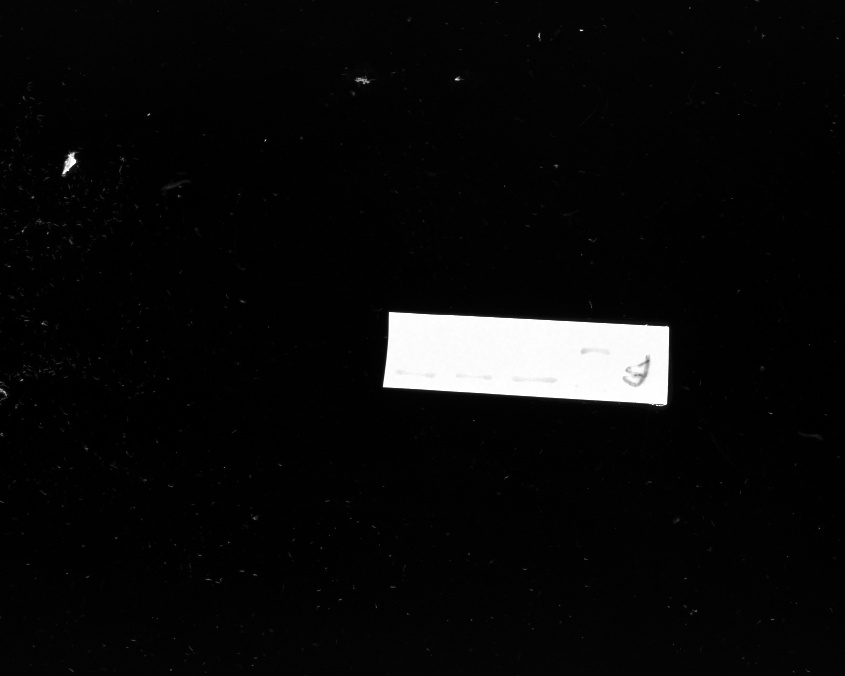

Supplement: Supplementary file 1 [file cancers-14-04645-s001.zip › cancers-1921606-original/westernblot_SPCA1_EMT/2021_01_14_actin/CHEMI_12232021_092323_(Membrane).jpg]

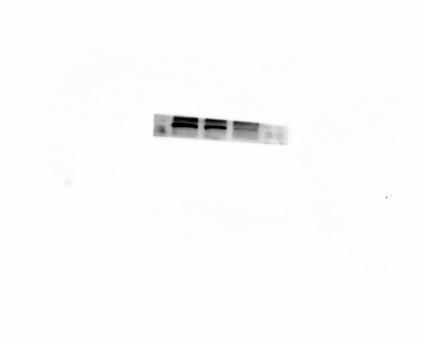

Supplement: Supplementary file 1 [file cancers-14-04645-s001.zip › cancers-1921606-original/westernblot_SPCA1_EMT/2021_01_14_nca/CHEMI_01142021_181121_(Chemi).jpg]

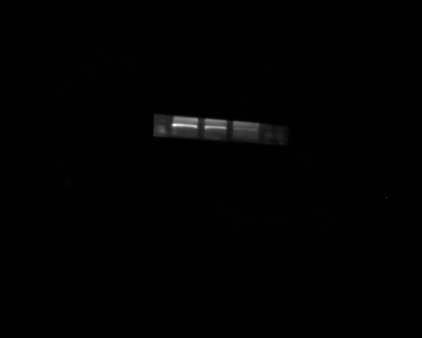

Supplement: Supplementary file 1 [file cancers-14-04645-s001.zip › cancers-1921606-original/westernblot_SPCA1_EMT/2021_01_14_nca/CHEMI_01142021_181121_(Chemi)_raw.tif]

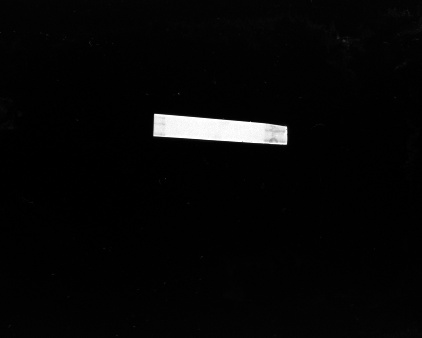

Supplement: Supplementary file 1 [file cancers-14-04645-s001.zip › cancers-1921606-original/westernblot_SPCA1_EMT/2021_01_14_nca/CHEMI_01142021_181121_(Membrane).jpg]

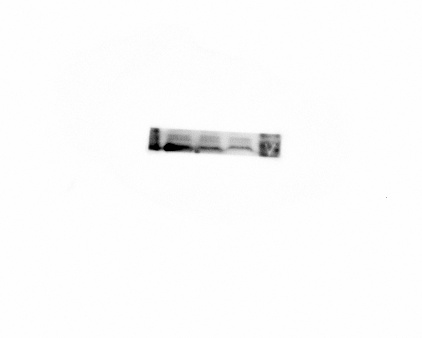

Supplement: Supplementary file 1 [file cancers-14-04645-s001.zip › cancers-1921606-original/westernblot_SPCA1_EMT/2021_01_14_vimentin/CHEMI_01142021_181905_(Chemi).jpg]

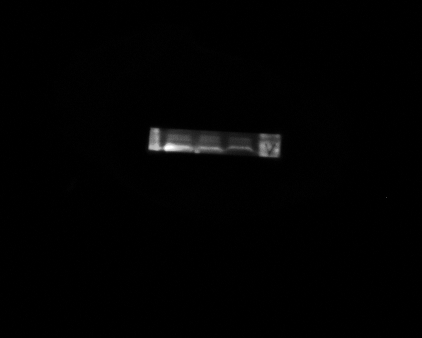

Supplement: Supplementary file 1 [file cancers-14-04645-s001.zip › cancers-1921606-original/westernblot_SPCA1_EMT/2021_01_14_vimentin/CHEMI_01142021_181905_(Chemi)_raw.tif]

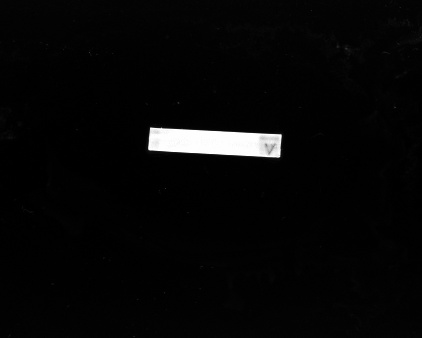

Supplement: Supplementary file 1 [file cancers-14-04645-s001.zip › cancers-1921606-original/westernblot_SPCA1_EMT/2021_01_14_vimentin/CHEMI_01142021_181905_(Membrane).jpg]

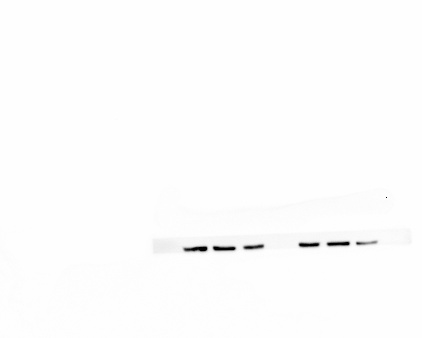

Supplement: Supplementary file 1 [file cancers-14-04645-s001.zip › cancers-1921606-original/westernblot_SPCA1_EMT/2021_01_25_actin/CHEMI_01252021_144922_(Chemi).jpg]

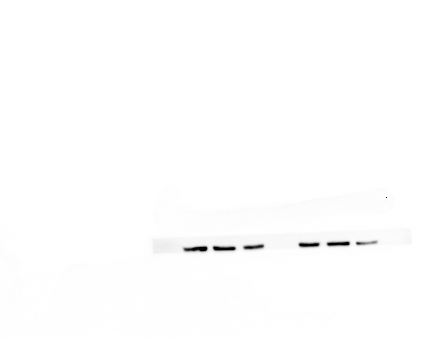

Supplement: Supplementary file 1 [file cancers-14-04645-s001.zip › cancers-1921606-original/westernblot_SPCA1_EMT/2021_01_25_actin/CHEMI_01252021_144922_(Chemi).tif]

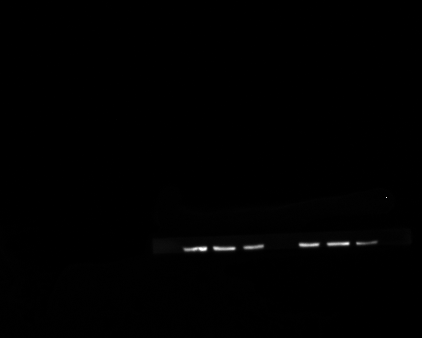

Supplement: Supplementary file 1 [file cancers-14-04645-s001.zip › cancers-1921606-original/westernblot_SPCA1_EMT/2021_01_25_actin/CHEMI_01252021_144922_(Chemi)_raw.tif]

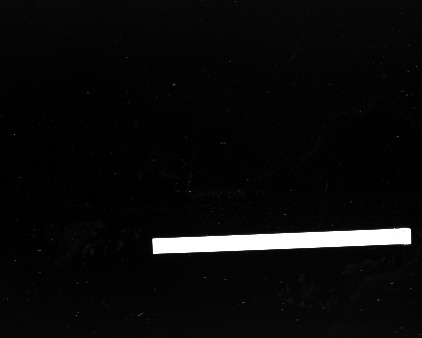

Supplement: Supplementary file 1 [file cancers-14-04645-s001.zip › cancers-1921606-original/westernblot_SPCA1_EMT/2021_01_25_actin/CHEMI_01252021_144922_(Membrane).jpg]

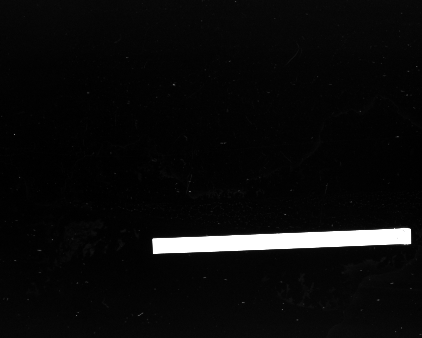

Supplement: Supplementary file 1 [file cancers-14-04645-s001.zip › cancers-1921606-original/westernblot_SPCA1_EMT/2021_01_25_actin/CHEMI_01252021_144922_(Membrane).tif]

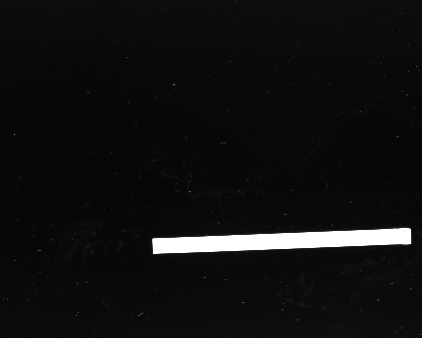

Supplement: Supplementary file 1 [file cancers-14-04645-s001.zip › cancers-1921606-original/westernblot_SPCA1_EMT/2021_01_25_actin/CHEMI_01252021_144922_(Membrane)_raw.tif]

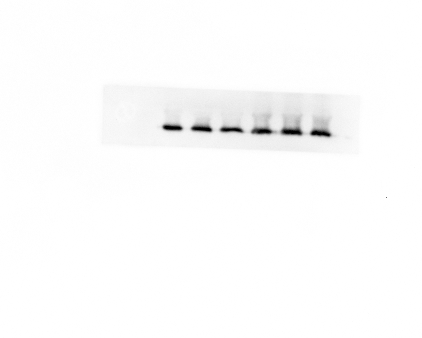

Supplement: Supplementary file 1 [file cancers-14-04645-s001.zip › cancers-1921606-original/westernblot_tumor tissue_apoptosis/full image_actin.tif]

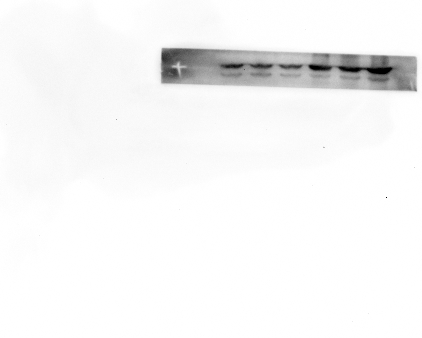

Supplement: Supplementary file 1 [file cancers-14-04645-s001.zip › cancers-1921606-original/westernblot_tumor tissue_apoptosis/full image_bax.tif]

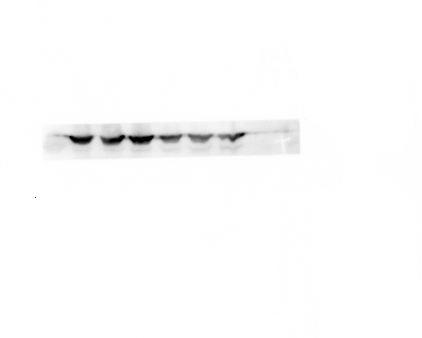

Supplement: Supplementary file 1 [file cancers-14-04645-s001.zip › cancers-1921606-original/westernblot_tumor tissue_apoptosis/full image_bcl2.tif]

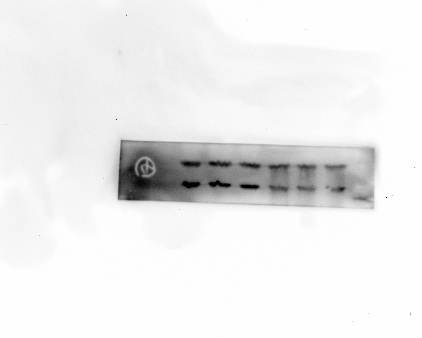

Supplement: Supplementary file 1 [file cancers-14-04645-s001.zip › cancers-1921606-original/westernblot_tumor tissue_apoptosis/full image_cas9.jpg]

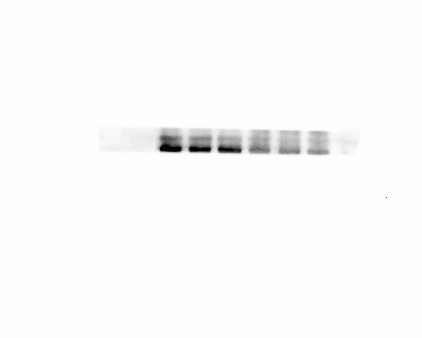

Supplement: Supplementary file 1 [file cancers-14-04645-s001.zip › cancers-1921606-original/westernblot_tumor tissue_apoptosis/full image_cytc.jpg]

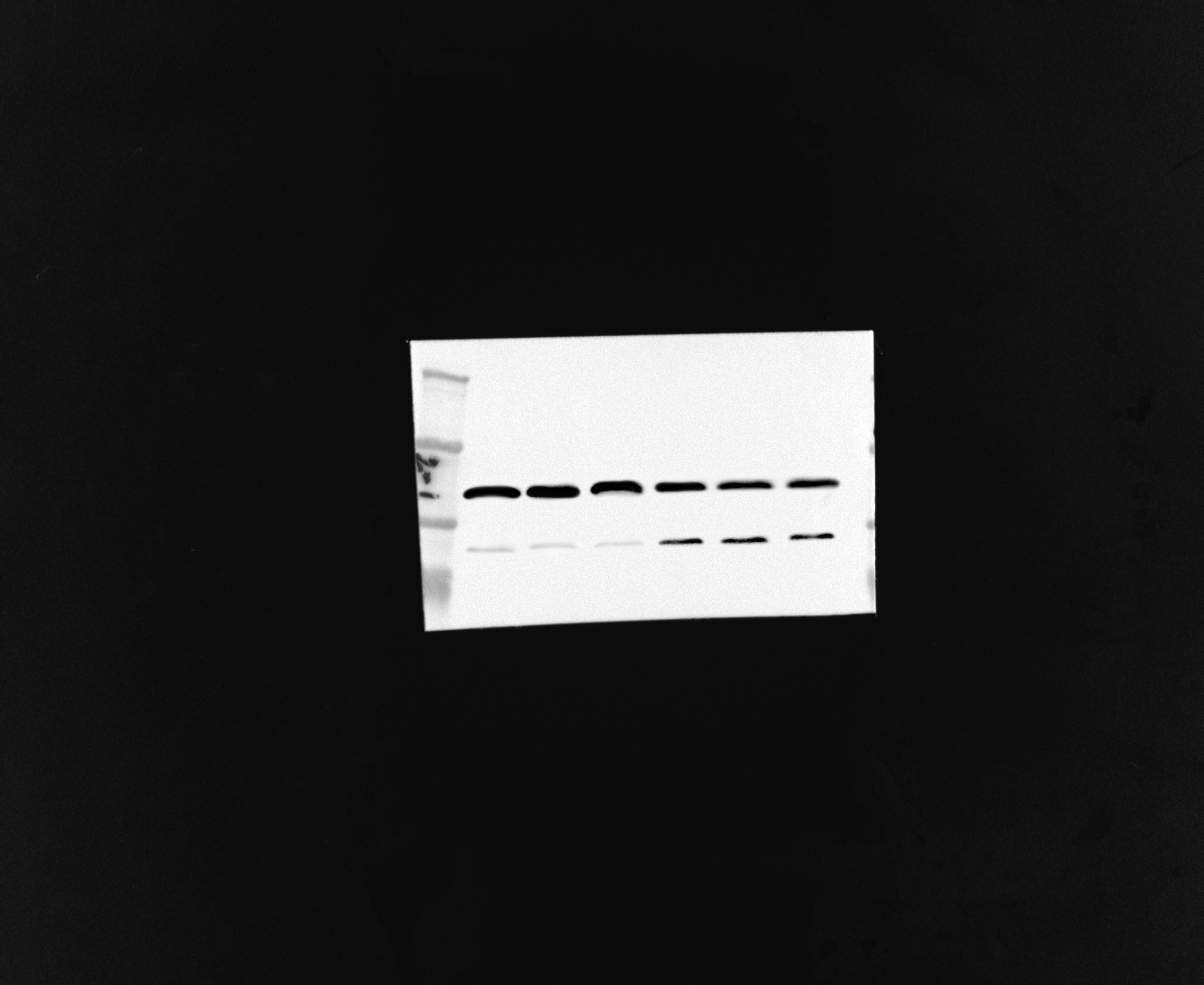

Supplement: Supplementary file 1 [file cancers-14-04645-s001.zip › cancers-1921606-original/westernblot_tumor tissue_apoptosis/full image_parp1.tif]

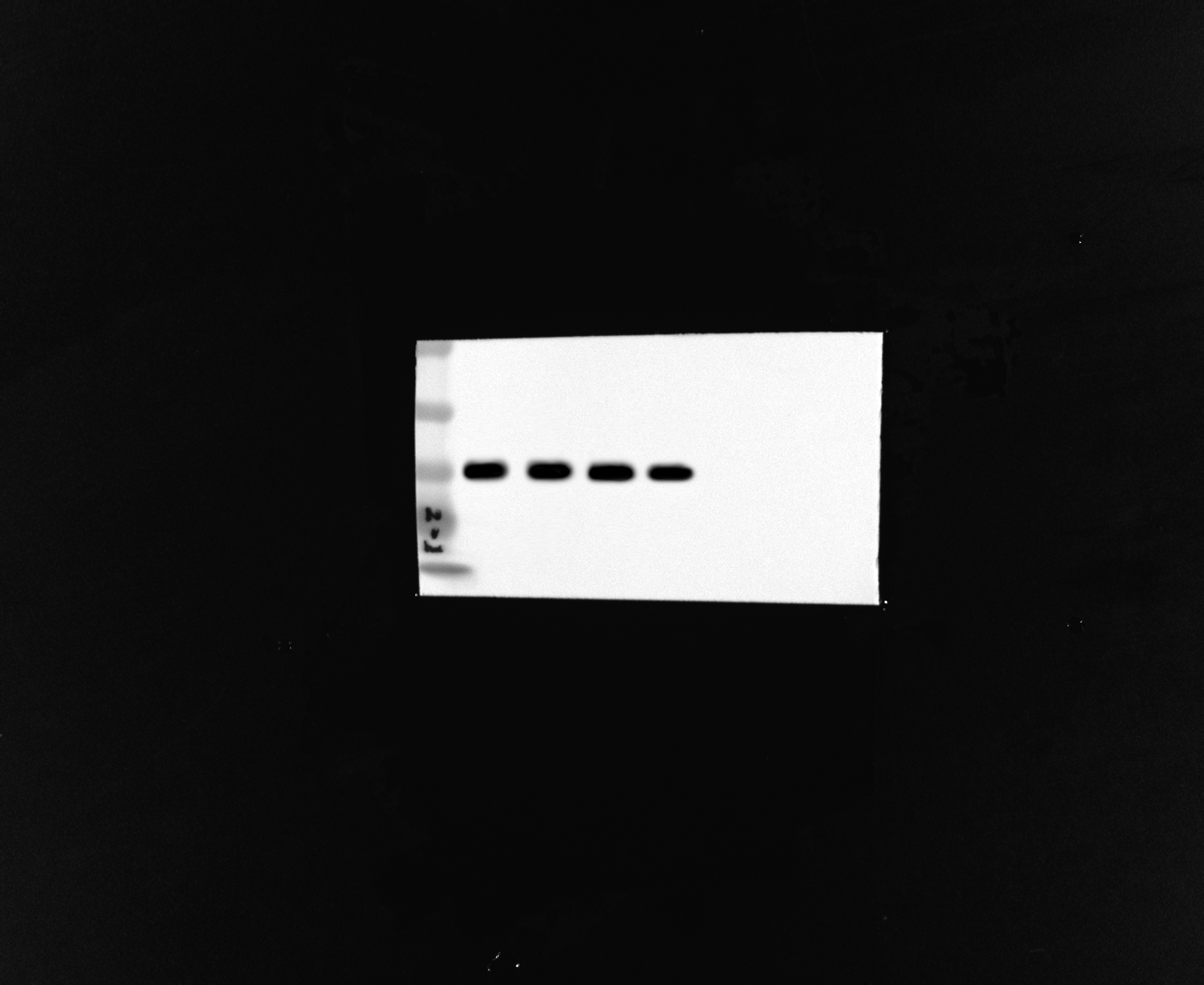

Supplement: Supplementary file 1 [file cancers-14-04645-s001.zip › cancers-1921606-original/westernblot_tumor tissue_EMT/Actin.tif]

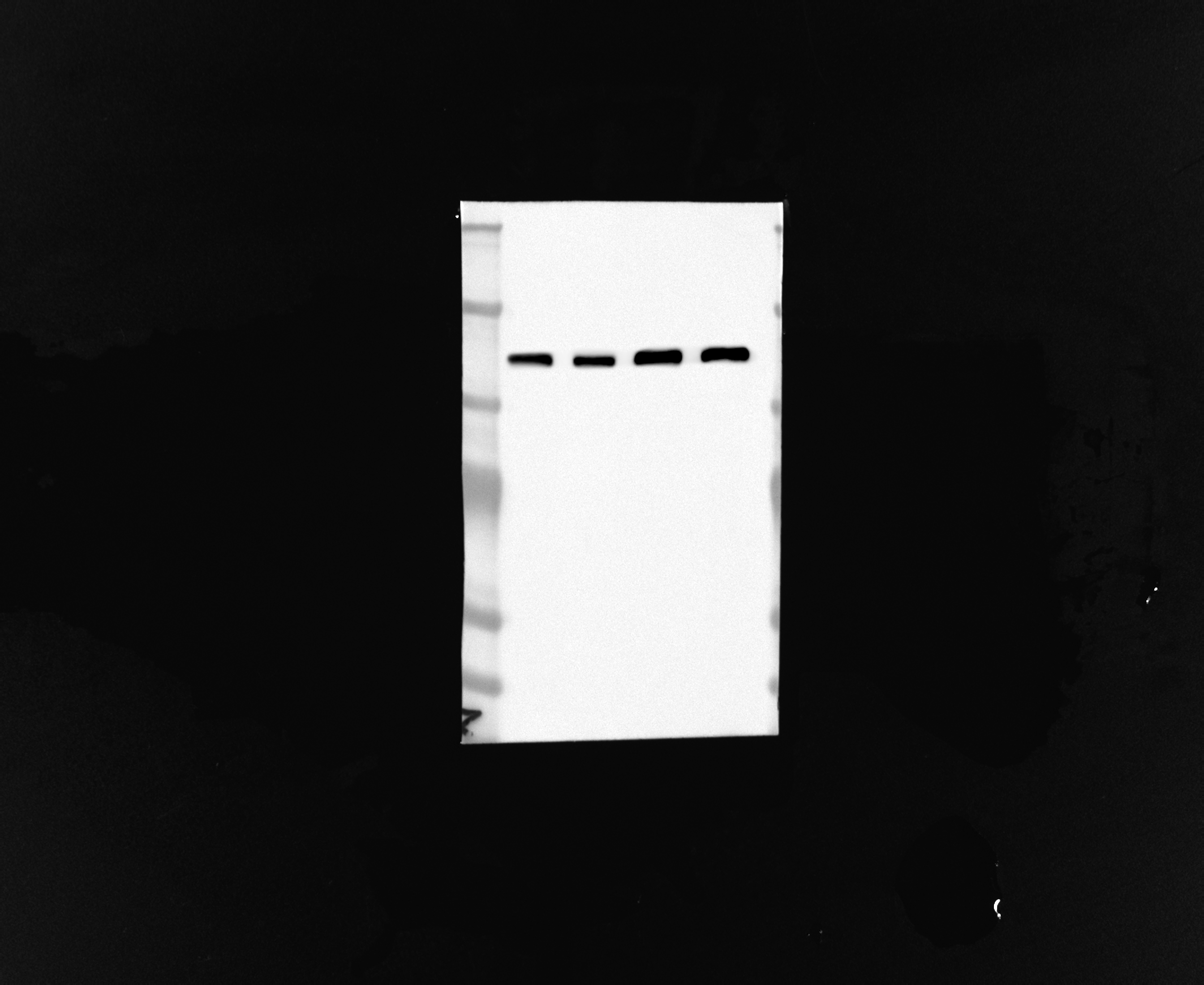

Supplement: Supplementary file 1 [file cancers-14-04645-s001.zip › cancers-1921606-original/westernblot_tumor tissue_EMT/E-cad.tif]

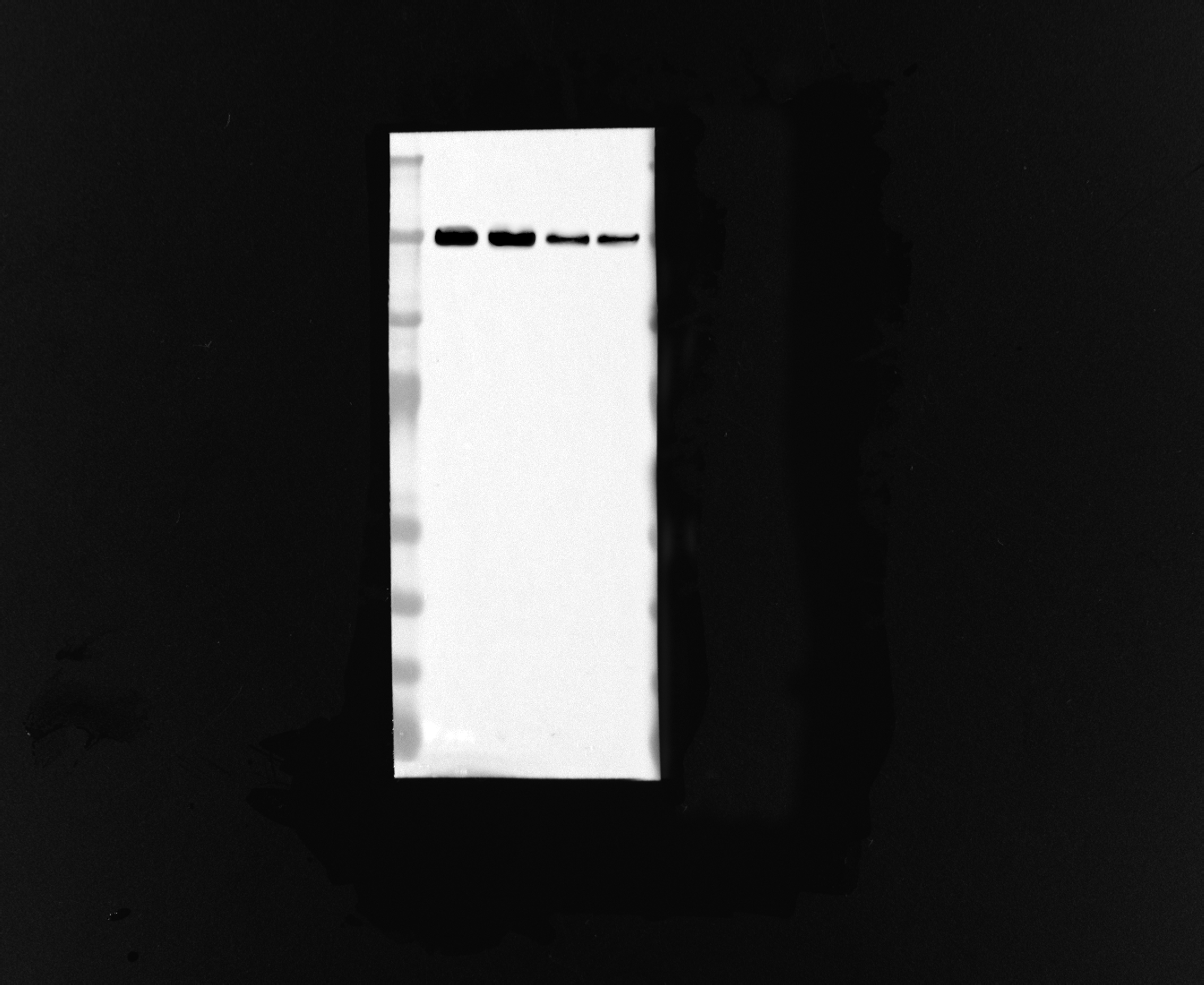

Supplement: Supplementary file 1 [file cancers-14-04645-s001.zip › cancers-1921606-original/westernblot_tumor tissue_EMT/N-cad.tif]

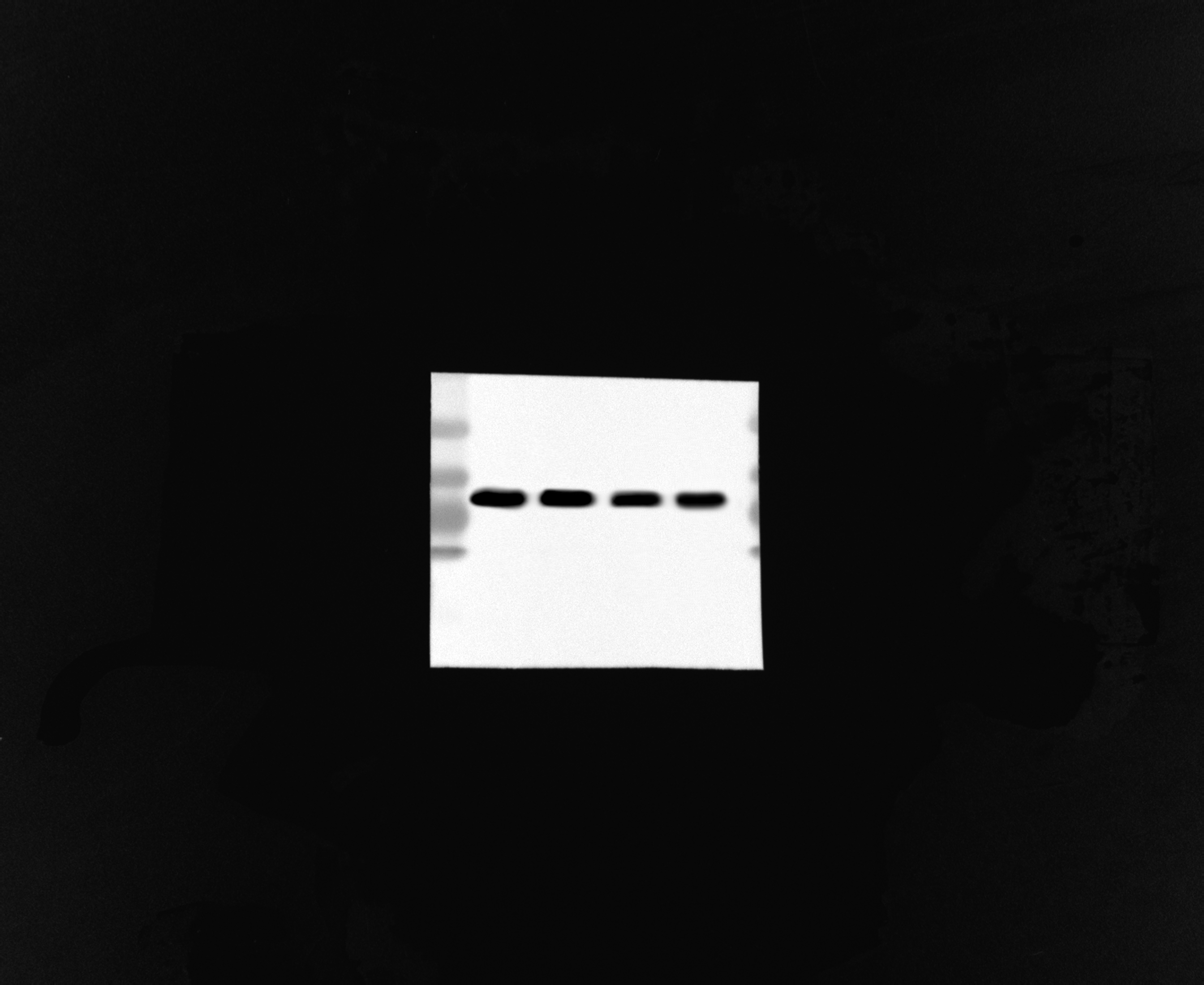

Supplement: Supplementary file 1 [file cancers-14-04645-s001.zip › cancers-1921606-original/westernblot_tumor tissue_EMT/slug.tif]

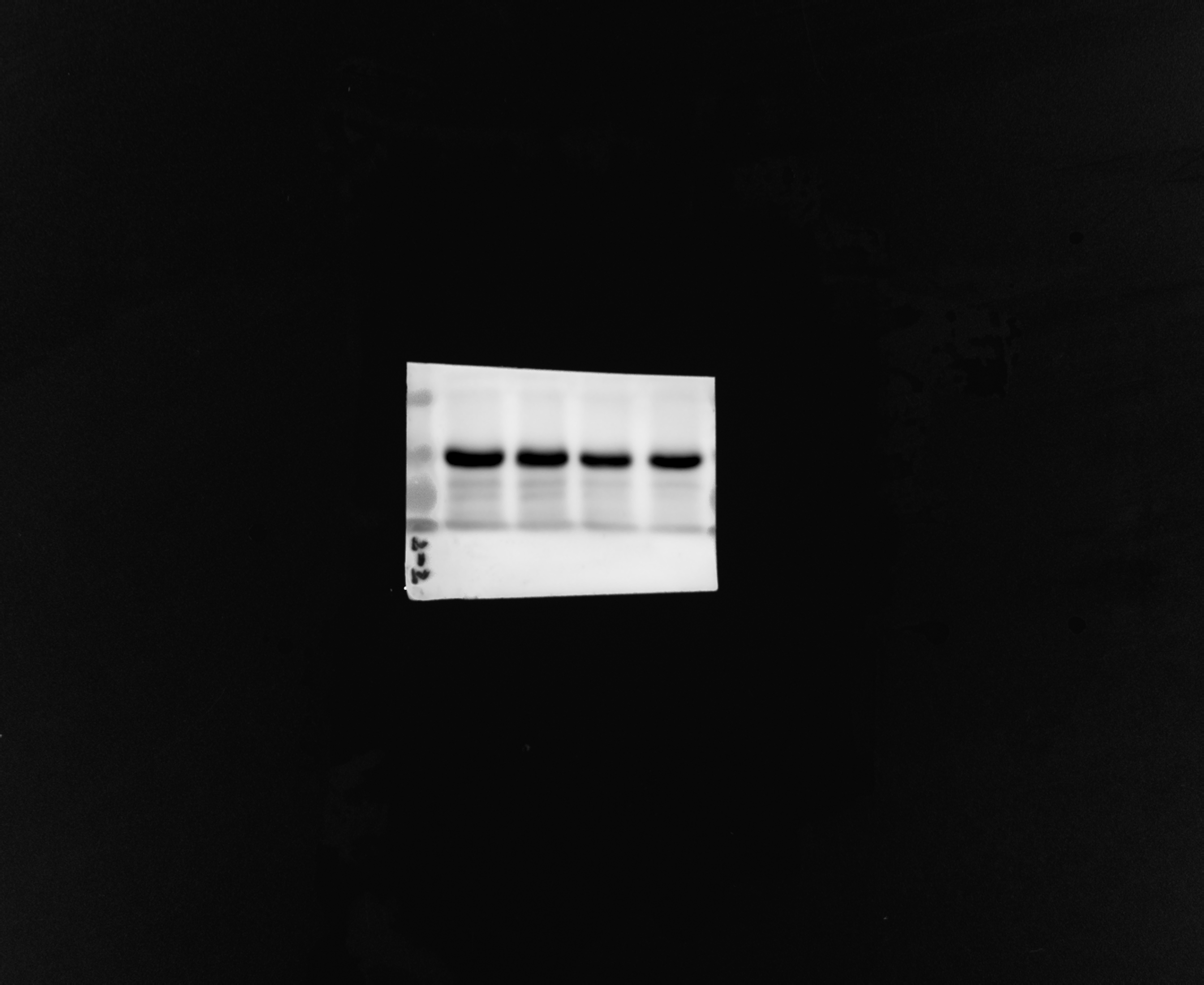

Supplement: Supplementary file 1 [file cancers-14-04645-s001.zip › cancers-1921606-original/westernblot_tumor tissue_EMT/snail.tif]
